# Construction of a lipid metabolism‐related and immune‐associated prognostic signature for hepatocellular carcinoma

**DOI:** 10.1002/cam4.3353

**Published:** 2020-08-19

**Authors:** Bo Hu, Xiao‐Bo Yang, Xin‐Ting Sang

**Affiliations:** ^1^ Department of Liver Surgery Peking Union Medical College Hospital Chinese Academy of Medical Sciences and Peking Union Medical College Beijing China

**Keywords:** hepatocellular carcinoma, immune system, lipid metabolism, prognosis

## Abstract

**Background:**

Hepatocellular carcinoma (HCC) is one of the most lethal malignancies. We aimed to identify a robust lipid metabolism‐related signature associated with the HCC microenvironment to improve the prognostic prediction of HCC patients.

**Methods:**

We analyzed the gene expression profiles of lipid metabolism from Molecular Signatures Database and information of patients from The Cancer Genome Atlas. Gene set variation analysis (GSVA), gene set enrichment analysis (GSEA), and principal component analysis (PCA) were employed for functional annotation. Quantitative real‐time polymerase chain reaction (qRT‐PCR) was employed to verify the expression of model genes in HCC and adjacent tissues.

**Results:**

As a result, a lipid metabolism‐related signature consisting of acyl‐CoA synthetase long‐chain family member 6 (*ACSL6*)*,* lysophosphatidylcholine acyltransferase 1*,* phospholipase A2 group 1B*,* lecithin‐cholesterol acyltransferase (*LCAT*), and sphingomyelin phosphodiesterase 4 (*SMPD4*) was identified among HCC patients. Lysophosphatidylcholine acyltransferase 1*, PLA2G1B,* and *SMPD4* were proved significantly high expression while *ACSL6* and *LCAT* were remarkably low expression in our 15 pairs of matched HCC and normal tissues by qRT‐PCR. Under different conditions, the overall survival (OS) of patients in low‐risk group was prolonged than that in high‐risk group. Moreover, the as‐constructed signature was an independent factor, which was remarkably associated with gender, histologic grade, and platelet level of HCC patients. In addition, the receiver operating characteristic (ROC) curve analysis confirmed the good potency of the model. Functional enrichment analysis further revealed that lower fatty acid (FA) oxidation and higher infiltration of immunocytes were detected in patients from the high‐risk group compared with those in the low‐risk group.

**Conclusions:**

Our findings indicate that the lipid metabolism‐related signature shows prognostic significance for HCC.

## Graphical abstract

Lipid signature was associated with transcription factor‐mediated network, lipid metabolism‐associated genes, and immune cell infiltration. Numerous papers have been focused on the alteration of lipid profile in hepatocellular carcinoma (HCC). Our study highlights the connection of altered lipid metabolism in microenvironment and immune system, which is an interesting topic nowadays. We propose an interesting high‐risk model based on five genes signature that further stratified in low‐ and high‐risk populations in Kaplan‐Meier curve. In addition, we suggest that aberrant lipid metabolism was related to increased gene profiles leading to fatty acid (FA) elongation and cellular response to FA that could shape the immunosuppressive environment of patients at high risk of HCC.

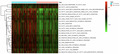

## 1 INTRODUCTION

Hepatocellular carcinoma (HCC) ranks the sixth place in terms of its morbidity, which also accounts for the main cause of cancer‐related deaths in the world.^1^ Hepatocellular carcinoma can be managed by diverse treatments, including chemotherapy, radiofrequency ablation, liver transplantation, and surgical treatment; however, its mortality is still high.^2^ Consequently, exploring the HCC features is urgently needed, so as to exploit novel therapies. Tumor microenvironment (TME) is acidic and hypoxic with nutrient deficiency, and it leads to aberrant metabolisms for tumor cells as well as those adjacent stromal cells, thus facilitating tumor metastasis, proliferation, and survival.^3^, ^4^ In HCC, its TME may show different metabolic disturbances, with lipid metabolic abnormality being the novel field, which has been attracting extensive interests in the last several years. Lipid metabolic disturbance, particularly for fatty acid (FA) metabolism with changed lipid‐metabolizing enzyme expression and activity due to the aberrantly activated oncogenic signaling pathways, has been increasingly identified to be the vital phenomenon of metabolic rewiring within immune cells and cancer cells, which might be involved in the development of HCC. Moreover, evidence obtained from some solid tumors suggests that, the tumor immunometabolic reprogramming is quite important, which is also identified as the new critical field for HCC research in the future.^5^ Emerging researches have suggested that immune cells play critical roles in the TME of HCC, and the abnormal lipid metabolism may significantly affect their functions and recruitment.^5^, ^6^ To date, few studies have combined lipid metabolism, immune status, and liver cancer progression, which is the core context of this study.

To this end, the present work aimed to shed more light on the possible significance of the lipid metabolism‐associated model in stratifying patient prognosis and its feasibility to guide therapeutic selection. Additionally, we also analyzed diverse immune statuses from the prognostic signature‐stratified patients in high‐ or low‐risk group. The prognostic model was constructed based on The Cancer Genome Atlas (TCGA) database, followed by further validation using the International Cancer Genome Consortium (ICGC) database.

## 2 MATERIALS AND METHODS

### 2.1 Data collection and mining of messenger RNA profiles

The level 3 messenger RNA expression patterns, together with related clinical information, were obtained based on 374 HCC as well as 50 healthy subjects derived from TCGA database (https://cancergenome.nih.gov). In this study, cases having ≤30 days of survival or those with no survival data were eliminated, since they might die of the fetal complications (including hemorrhage, intracranial infection, and heart failure HF) but not HCC. Afterward, we used the “caret” of R package to ^7^ randomly classify the 343 HCC samples into training set (n = 172) or test set (n = 171) for later analyses. Patients in training set were used to construct the prognostic immune gene signature, while those in test set were used to validate the prediction efficiency for the constructed signature. Ethical approval was exempted from this study, since all data used were available in public database.

In addition, we collected 183 lipid metabolism‐associated genes based on the Molecular Signatures Database v7.0.^8^, ^9^ (c2: KEGG gene sets) (Fatty acid metabolism M699, Glycerophospholipid metabolism M9131, Glycerolipid metabolism M15902, Sphingolipid metabolism M15955, Ether lipid metabolism M2130, Glycosphingolipid biosynthesis‐ganglio series M8535, Biosynthesis of unsaturated FAs M11673, Glycosphingolipid biosynthesis‐globo series M12899, Glycosphingolipid biosynthesis‐lacto, and neolacto series M17377; http:// www.broadinstitute.org/gsea/msigdb/index.jsp). Afterward, differentially expressed genes (DEGs) were identified using the edgeR algorithm for subsequent analyses (*P* < .05, log FC > 1; FC, fold change).

### 2.2 Transcription factors extraction and regulatory network construction

Survival‐associated metabolic genes were selected by univariate Cox regression using survival package of R software, and a difference of *P* < .05 indicated statistical significance. For better investigating the interactions between these selected genes in the context of HCC, we established a transcription factor (TF)‐mediated network. Transcription factors have been recognized as the vital molecules for the direct control of gene expression, and Cistrome Cancer represents the data source integrating the TCGA cancer genome data with more than 23 000 ChIP‐seq as well as chromatin accessibility profiles, and it offers regulatory connections of TFs with transcriptomes by covering a total of 318 TFs.^10^ Usually, for transcriptional data, differential genes are used to compare with TFs for identifying TFs with differential expression and for plotting volcano map and heatmap. In addition, differential TFs may be related to survival‐associated metabolic genes together with a mapping regulatory network by the use of Cytoscape 3.7.2.^11^

### 2.3 Prognostic lipid metabolism‐related signature construction and validation

We conducted univariate and multivariate Cox regression analyses, together with Least Absolute Shrinkage and Selection Operator (LASSO) analysis, for constructing the prognostic lipid metabolism‐related model, aiming at predicting the OS of HCC patients. Least Absolute Shrinkage and Selection Operator has been developed as a penalized regression employing the L1 penalty for shrinking regression coefficient to zero, so as to eliminate multiple variables on the basis of the selection of fewer predictors with the greater penalty. Based on LASSO analysis, those key genes were extracted from the as‐mentioned survival‐related metabolic genes. Additionally, prognostic contributions by all genes were evaluated by multivariate Cox regression analysis.

For confirming the value of that constructed signature in predicting prognosis, we utilized the “survminer” and “survival” R packages for plotting Kaplan‐Meier (K‐M) survival curves. In addition, we used time‐dependent receiver operating characteristic curve in assessing our model efficiency in predicting prognosis across three groups by determining area under the curve (AUC) values.^12^ We conducted univariate as well as multivariate Cox proportional hazard regression analysis for confirming the signature efficiency in independent prognosis prediction based on the conventional clinical parameters, such as gender, age, status of hepatitis virus infection, clinical stage, portal vein invasion, bile duct invasion, vein invasion, along with hepatic fibrosis of ICGC HCC cohort. Moreover, we also performed PCA for dimensionality reduction, thus identifying various synthetic parameters for exploring the grouping ability of our model. PCA can be used as the statistical approach for determining critical parameters within the multidimensional data set, and it can explain observational heterogeneities and is thereby adopted for simplifying analysis and visualizing multidimensional data sets.^13^ In this study, we used limma^14^ and scatterplot3d^15^ packages to implement PCA.

### 2.4 Gene set variation analysis

To further detect the distinct lipid metabolism between low‐ and high‐risk groups that was derived from our signature, we subsequently conducted the gene set variation analysis (GSVA), the approach for gene set enrichment to unsupervised estimate pathway activity variations among certain population.^16^ After screening, 115 Gene Oncology (GO) items related to lipid metabolism were incorporated into the calculation via R package “GSVA”, while *P* < .05 and logFC > 0.1/<−0.2 were considered statistically significant.

### 2.5 Relationships of the prognostic lipid metabolism‐related signature with immune cell infiltration

A body of previous literature has demonstrated that lipid metabolic reprogramming has a bearing on the immune cells and tumor progression,^17^, ^18^ which allows us to further explore the relationship between the constructed prognostic model and immune cell infiltration in the context of HCC. The online database Tumor IMmune Estimation Resource (TIMER) serves as an online resource for the systemic evaluation of clinical significance for different immunocytes to different types of cancers, and it can be used to analyze and visualize the tumor‐infiltrating immunocyte levels.^19^ In TIMER, a total of 10 009 TCGA samples from 23 types of cancers are enrolled for estimating the contents of six subtypes of tumor‐infiltrating immunocytes, such as CD4 T cells, CD8 T cells, B cells, macrophages, dendritic cells, and neutrophils. As a result, TIMER is adopted for determining the association of immunocyte infiltration with additional factors. In the present work, we downloaded the immune infiltrating degrees for patients with HCC, and then calculated associations between the immunocyte infiltrating levels and our prognostic signature.

Moreover, gene set enrichment analysis (GSEA) was also used to assess the different immune statuses in low‐ compared with high‐risk groups screened by our model. Gene set enrichment analysis, the calculation method for determining the statistical significance of the previously defined gene set, as well as the concordant heterogeneities of two biological statuses.^8^ Typically, the false discovery rate *q* < 0.25 and *P* < .05 were selected as the significance thresholds. After 1000 permutations, we presented those six most significant gene sets with the highest normalized enrichment scores (NES). ESTIMATE^20^ was also adopted to measure the immune cell infiltration level (immune score), tumor purity, and stromal content (stromal score) for respective HCC sample.

### 2.6 Quantitative real‐time polymerase chain reaction validation of the expression of the model genes

According to the results of bioinformatics analysis, the expression level of genes that were included in the lipid metabolism‐related signature was validated by quantitative real‐time polymerase chain reaction (qRT‐PCR) using 15 pairs of matched HCC and para‐carcinoma tissue obtained from our center. Total RNA was extracted from tissues using TRIzol reagent (Nanjing Biosky Inc) and reverse transcribed into cDNA using reverse transcriptase (Nanjing Biosky Inc) according to the manufacturer's instructions. The qRT‐PCR reaction was performed using real‐time PCR kit (Nanjing Biosky Inc) on a CFX384 Touch Real‐Time PCR Detection System (Bio‐Rad Inc). The qRT‐PCR amplification program was set as follows: 95°C for 3 minutes, followed by 40 cycles of 95°C for 30 seconds and 55°C for 20 seconds, and finally 72°C for 20 seconds. After completing the amplification reaction, a melt curve of PCR product was plotted (95°C for 15 seconds; 60°C for 15 seconds; and 95°C for 15 seconds). The expression level of model genes was normalized to the internal control GAPDH and calculated according to the 2^−ΔΔCT^ method.

### 2.7 Statistical methods

Statistical analysis was conducted by R (v.3.6.1) software. In addition, Fisher's exact test or Pearson *χ* ^2^ test was utilized for exploring the qualitative variables. A difference of *P* < .05 indicated statistical significance.

## 3 RESULTS

### 3.1 Differentially expressed survival‐associated metabolic gene mining and TF regulatory network establishment

**FIGURE 1:**
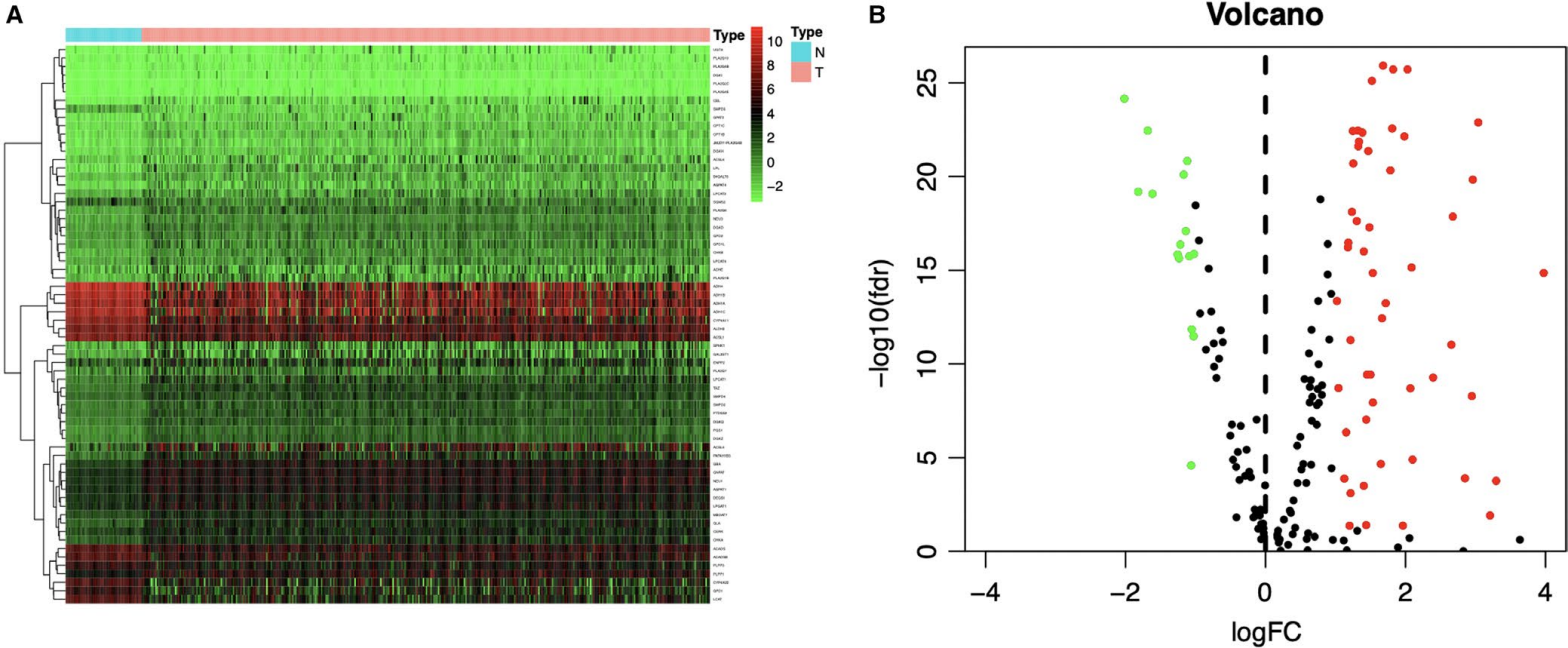
Differentially expressed lipid metabolism‐related genes in The Cancer Genome Atlas hepatocellular carcinoma (HCC) patients. Heatmap (A) and volcano plot (B) demonstrate differentially expressed lipid metabolism‐related genes between HCC and non‐tumor tissues. In terms of the heatmap, the colors from green to red represent low to high gene expression levels. In the volcano plot, red dots represent differentially upregulated expressed genes, green dots represent differentially downregulated expressed genes, and black dots represent no differentially expressed genes. N, normal tissue. T, tumor

**FIGURE 2:**
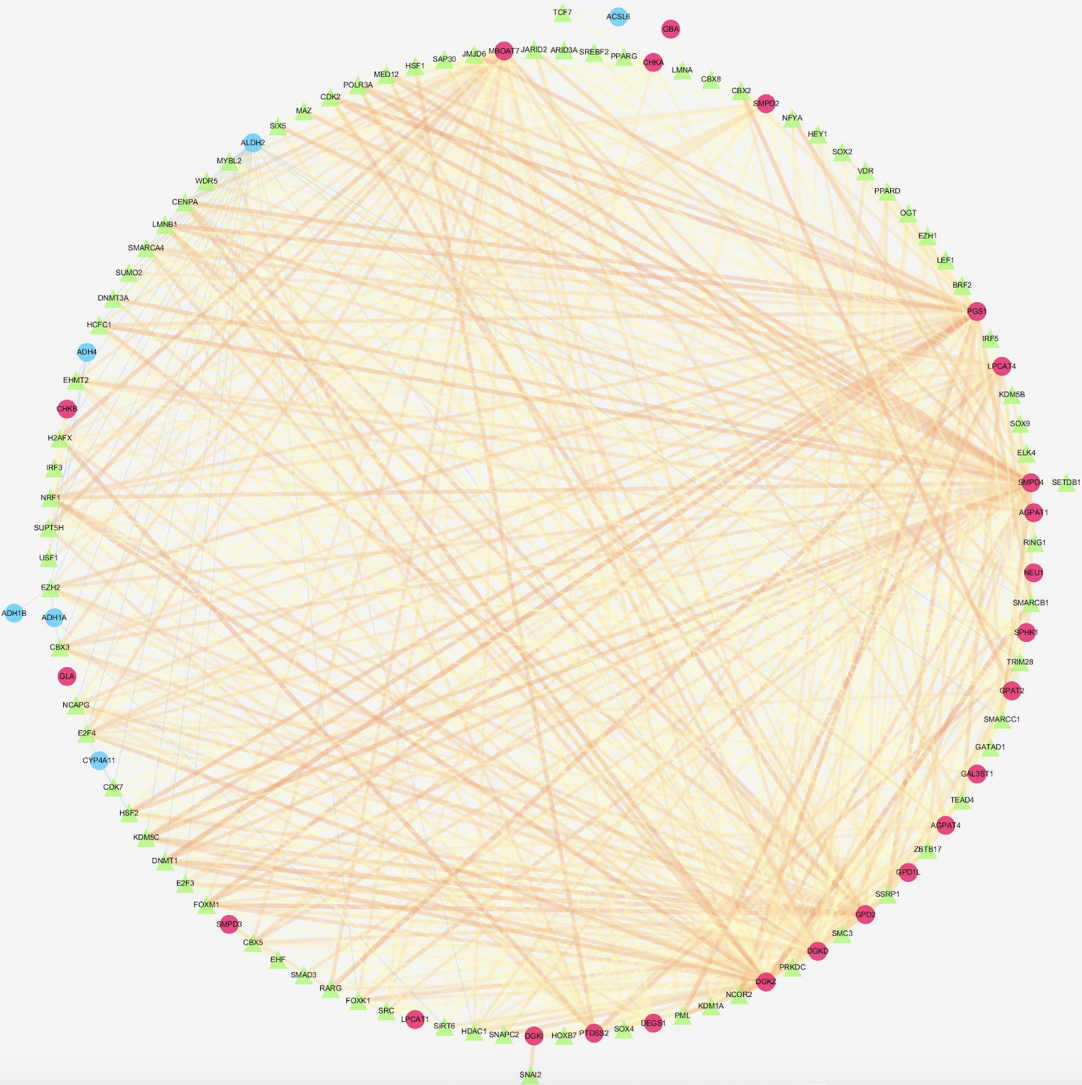
Transcription factor (TF)‐mediated regulatory network. Regulatory network constructed based on differently expressed TFs and survival‐relevant genes. The red circle represents high‐risk genes, and the blue circle represents low‐risk genes. The green triangle represents transcription factors, and the thickness and brightness of lines between nodes represent the level of relevance (low Cor values to small sizes and dark colors)

In the primary screening by edgeR algorithm, a total of 66 metabolic genes were identified as DEGs for further analyses, among which, 51 were upregulated, whereas 15 were downregulated (*P* < .05, logFC > 1) (Figure 1A,B). We further examined the regulatory mechanisms for the abovementioned genes. To be specific, we determined expression profiles for altogether 318 TFs. As a result, 116 TFs showed differential expression between the TCGA‐derived HCC cohort and the normal liver samples (Figure S1A,B). Then, we also investigated those possible relationship between such TFs with differential expression and those prognostic lipid metabolism‐related genes. As a result, 116 TFs and 65 metabolic genes were selected to construct the regulatory networks, according to the Pearson correlation coefficient of >0.4 and the *P* < .001 (Figure 2).

**FIGURE 3:**
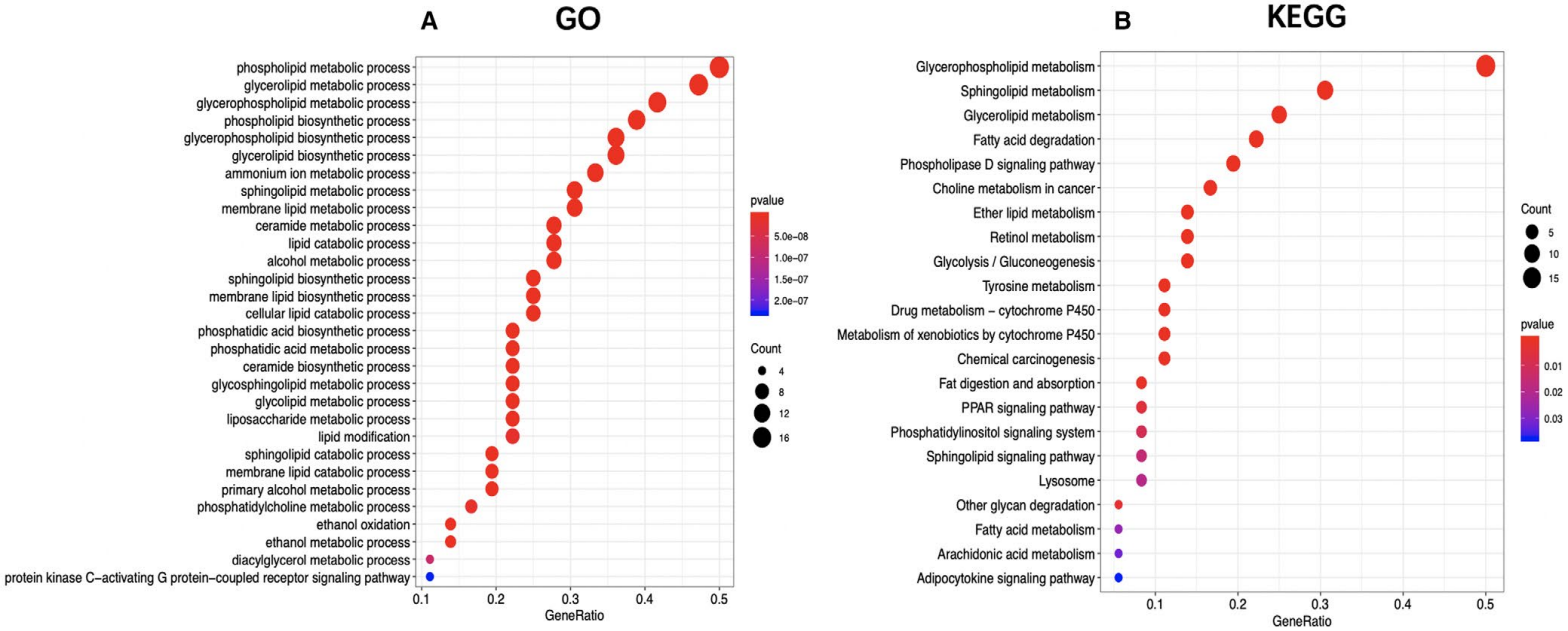
The Gene Ontology (GO) and Kyoto encyclopedia of genes and genomes (KEGG) analysis of the survival‐related lipid metabolism‐associated genes in hepatocellular carcinoma

Moreover, we identified 36 genes with significant correlation with the overall survival (OS) for TCGA‐derived HCC cases (*P* < .05) (Table S1). The potential functions of these genes were ascertained by GO along with Kyoto encyclopedia of genes and genomes (KEGG) pathway analyses. Not surprisingly, several lipid metabolism‐related GO terms were identified in the biological process, like “Lipid catabolic process,” “Lipid modification,” and “Phospholipid metabolic process” (Figure 3A). In addition, “Mitochondrial outer membrane,” “Organelle outer membrane,” and “Outer membrane” were the most significantly enriched GO items of cellular component (Figure S2A). Furthermore, the top 3 GO terms of molecular function (MF) were “O‐acyltransferase activity,” “Transferase activity, transferring acyl groups other than amino‐acyl groups,” and “Transferase activity, transferring acyl groups” (Figure S2B). As indicated from the KEGG pathway analysis, the lipid metabolism‐related genes were mostly enriched via several pathways, including “Fatty acid metabolism,” “PPAR signaling pathway,” and “Choline metabolism in cancer” (Figure 3B).

### 3.2 Risk score signature establishment and predicting efficiency assessment

**FIGURE 4:**
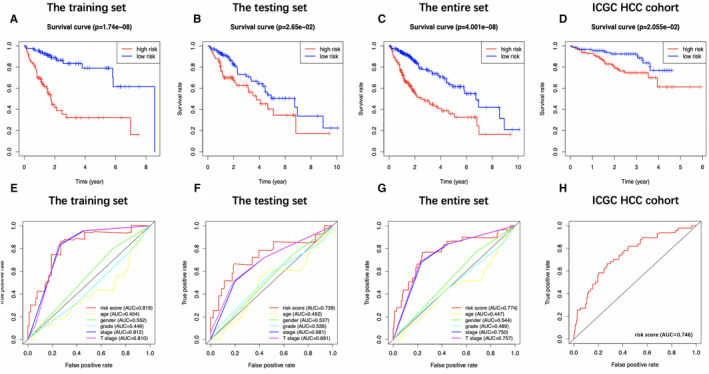
Prognostic analysis of the risk score signature. Kaplan‐Meier survival curves of patients in high‐risk group and low‐risk group of training set (A), testing set (B), entire set (C), and the International Cancer Genome Consortium database hepatocellular carcinoma (ICGC HCC) cohort (D) are shown. Patients in high‐risk group suffered shorter overall survival than those in low‐risk group. (E)‐(H) show survival‐dependent receiver operating characteristic curves validation at 1 y of prognostic value of the prognostic index in the four sets (the training set, the testing set, the entire set, and the ICGC HCC cohort, respectively)

**FIGURE 5:**
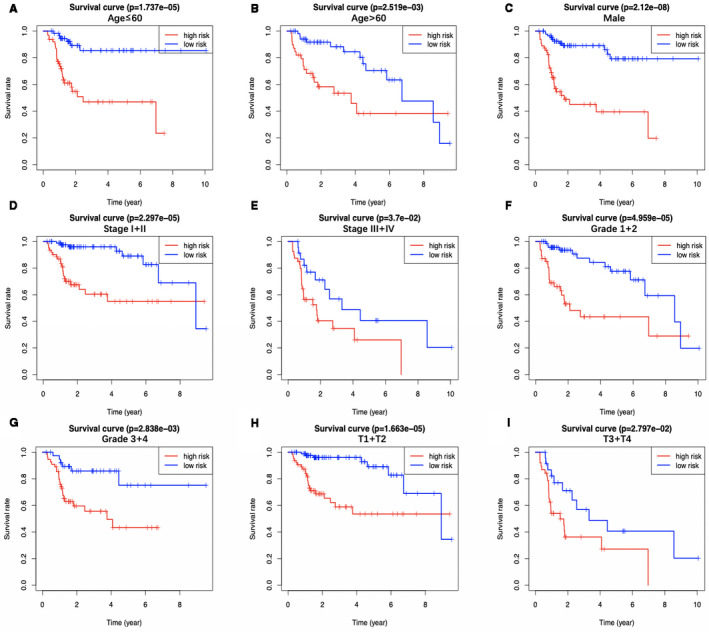
The overall survival differences between the high‐risk group and the low‐risk group from The Cancer Genome Atlas are shown under the conditions of classifying patients by age (A, B), male (C), clinical stage (D, E), tumor grade (F, G), and T stage (H, I). Low‐risk patients display longer overall survival than high‐risk patients

**FIGURE 6:**
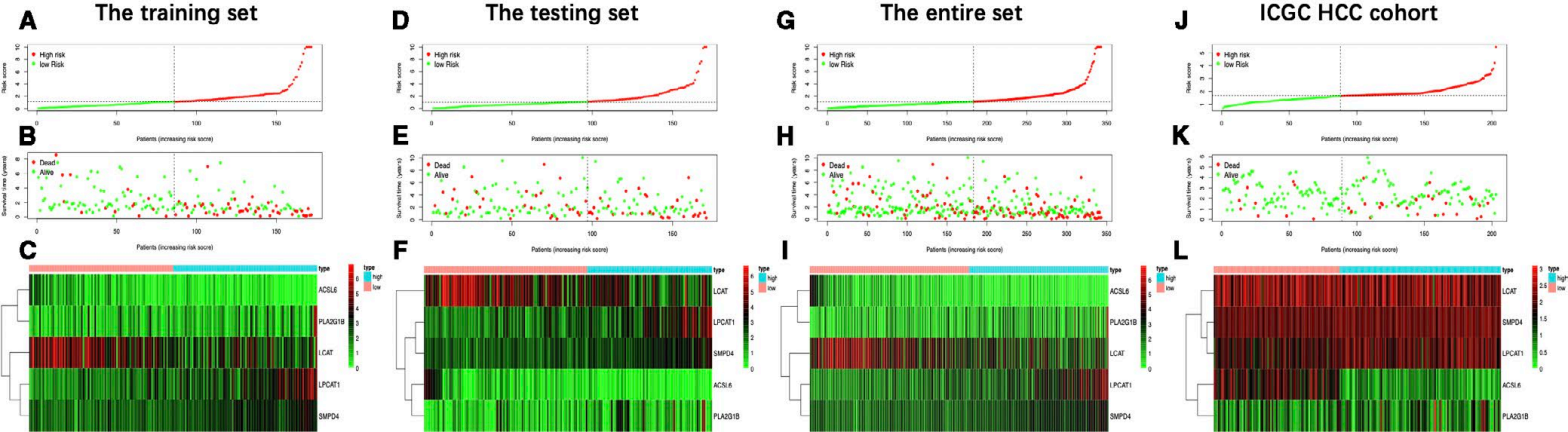
Distribution of risk score, overall survival, gene expression in the training set (A‐C), the testing set (D‐F), the entire set (G‐I), and the International Cancer Genome Consortium database hepatocellular carcinoma (ICGC HCC) cohort (J‐L). Distribution of risk score, overall survival, and heatmap of the expression of five signature genes in low‐risk and high‐risk groups is listed in the picture from top to bottom

Five metabolic genes, acyl‐CoA synthetase long‐chain family member 6 (ACSL6), phospholipase A2 group 1B (PLA2G1B), sphingomyelin phosphodiesterase 4 (SMPD4), lysophosphatidylcholine acyltransferase 1 (LPCAT1), and lecithin‐cholesterol acyltransferase (LCAT), were subsequently selected from the 36 OS‐related genes via LASSO and multivariate Cox regression analyses to establish a prognostic model, aiming to categorize HCC patients into two groups with discrete OS, namely, the high‐ and low‐risk groups (Table 1). Risk score was calculated by the following formula: Risk score = [ACSL6 expression*(−0.4665)] + [LPCAT1 expression*(0.0321)] + [PLA2G1B expression*(0.0541)] + [LCAT expression*(−0.0219)] + [SMPD4 expression*(0.1444)]. Based on the median risk score, all cases were classified as high‐ or low‐risk group. According to the K‐M analysis (Figure 4A‐D), high‐risk patients showed remarkably reduced OS relative to low‐risk patients among diverse sets. Additionally, with regard to 1‐year OS, the AUC values for training set, test set, entire set, and the ICGC HCC cohort were 0.818, 0.739, 0.774, and 0.746, respectively, which suggested the good performance of the risk score signature (Figure 4E‐H). Our established signature had the best AUC value relative to those clinicopathological characteristics of TCGA cases, which also reflected that it had remarkable predicting efficacy. Moreover, we also investigated those prognostic effects of high‐ or low‐risk group using the same clinical parameters. For the entire TCGA set, low‐risk patients had remarkably better prognosis than high‐risk patients in terms of subgroups stratified by age (≤60/>60; Figure 5A,B), male (Figure 5C), stage (I + II/III + IV; Figure 5D,E), grade (G1 + G2/G3 + G4; Figure 5F,G), as well as T stage (T1 + T2/T3 + T4; Figure 5H,I) (*P* < .05). For better exploring whether the lipid metabolism‐related model was able to predict prognosis independently, we conducted univariate as well as multivariate analysis. As a result, the risk score might serve as the indicator to independently predict prognosis (Tables 2 and 3). In addition, we plotted the distribution of risk score, survival status as well as gene expression in lipid metabolism‐related signature of training set (Figure 6A‐C), test set (Figure 6D‐F), entire set (Figure 6G‐I), and the ICGC HCC cohort (Figure 6J‐L), respectively.

**TABLE 1:** Five lipid metabolism‐related signature genes identified from Cox regression analysis from TCGA

ID	Coef	HR	HR.95L	HR.95H
ACSL6	−0.4664998	0.62719376	0.42768696	0.91976621
LPCAT1	0.03213162	1.03265341	1.00667644	1.05930071
PLA2G1B	0.05411254	1.0556034	1.02047958	1.09193614
LCAT	−0.021873	0.97836445	0.95687081	1.00034089
SMPD4	0.14443333	1.15538467	1.05089409	1.27026475

Abbreviations: Coef, coefficient; H, high; HR, hazard ratio; L, low; TCGA, The Cancer Genome Atlas.

**TABLE 2:** Univariate and multivariate Cox regression analyses of clinicopathologic characteristics associated with overall survival in The Cancer Genome Atlas samples

Variable	The training set				The testing set				The entire set			
	Univariate analysis		Multivariate analysis		Univariate analysis		Multivariate analysis		Univariate analysis		Multivariate analysis	
	HR (95% CI)	*P‐*value	HR (95% CI)	*P‐*value	HR (95% CI)	*P‐*value	HR (95% CI)	*P‐*value	HR (95% CI)	*P‐*value	HR (95% CI)	*P‐*value
Age (>60/≤60)	0.999 (0.967‐1.032)	.954	—	—	1.039 (1.004‐1.076)	.027	1.057 (1.014‐1.102)	.009^a^	1.018 (0.995‐1.041)	.125	—	—
Gender (male/female)	0.445 (0.203‐0.976)	.043^a^	0.557 (0.222‐1.394)	.211	0.883 (0.393‐1.982)	.762	—	—	0.601 (0.345‐1.048)	.073	—	—
Grade (G4/G3/G2/G1)	1.885 (1.168‐2.584)	.009^a^	2.76 (1.423‐5.368)	.003^a^	0.967 (0.529‐1.768)	.914	—	—	1.458 (0.997‐2.133)	.052	—	—
Stage (IV/III/II/I)	1.733 (1.162‐2.584)	.007^a^	2.945 (1.112‐7.776)	.029^a^	1.445 (0.866‐2.411)	.158	—	—	1.595 (1.184‐2.148)	.002^a^	2.479 (0.953‐6.450)	.063
T stage (T4/T3/T2/T1)	1.56 (1.051‐2.316)	.028^a^	0.691 (0.275‐1.733)	.431	1.506 (0.923‐2.458)	.102	—	—	1.524 (1.134‐2.047)	.005^a^	0.647 (0.250‐1.673)	.369
Albumin (>3.5/≤3.5 g/dL)	0.999 (0.983‐1.015)	.9	—	—	0.825 (0.598‐1.138)	.241	—	—	0.964 (0.740‐1.255)	.784	—	—
Platelet (>250/≤250 × 10^9^/L)	0.999 (0.998‐1.000)	.625	—	—	1.000 (0.999‐1.001)	.941	—	—	0.999 (0.998‐1.001)	.288	—	—
AFP (>20/≤20 ng/mL)	1 (0.999‐1.002)	.329	—	—	0.998 (0.993‐1.003)	.584	—	—	0.999 (0.997‐1.001)	.435	—	—
Vascular invasion (macro/micro/none)	1.693 (0.897‐3.190)	.103	—	—	2.080 (1.190‐3.634)	.01^a^	3.768 (1.778‐7.984)	<.001^a^	1.920 (1.263‐2.920)	.002^a^	1.962 (1.223‐3.144)	.005^a^
Risk score	1.632 (1.284‐2.075)	<.001^a^	1.507 (1.134‐2.002)	.005^a^	1.180 (1.019‐1.366)	.03^a^	1.289 (1.046‐1.590)	.017^a^	1.257 (1.125‐1.406)	<.001^a^	1.201 (1.054‐1.370)	.006^a^

Abbreviations: AFP, alpha‐fetoprotein; CI, confidence interval; HR, hazard ratio.

^a^ Statistically significant.

**TABLE 3:** Univariate and multivariate Cox regression analyses of clinicopathologic characteristics associated with overall survival in the International Cancer Genome Consortium

Variable	Univariate analysis		Multivariate analysis	
	HR (95% CI)	*P‐*value	HR (95% CI)	*P‐*value
Age (>60/≤60)	1.021 (0.985‐1.057)	.255	—	—
Gender (male/female)	1.186 (0.538‐2.617)	.673	—	—
Hepatitis virus (none/infection)	0.929 (0.360‐2.394)	.878	—	—
Stage (IV/III/II/I)	1.373 (0.927‐2.035)	.114	—	—
Portal vein invasion (none/invasion)	1.076 (0.694‐1.671)	.741	—	—
Vein invasion (none/invasion)	2.243 (1.296‐3.882)	.004^a^	2.160 (0.991‐4.708)	.052
Bile duct invasion (none/invasion)	0.481 (0.066‐3.516)	.471	—	—
Fibrosis (none/fibrosis)	1.428 (0.195‐10.439)	.726	—	—
Risk score	6.227 (3.601‐10.770)	<.001^a^	7.378 (3.850‐14.145)	<.001^a^

Abbreviations: CI, confidence interval; HR, Hazard ratio.

^a^ Statistically significant.

### 3.3 Principal component analysis validated the stratification capacity of signature

**FIGURE 7:**
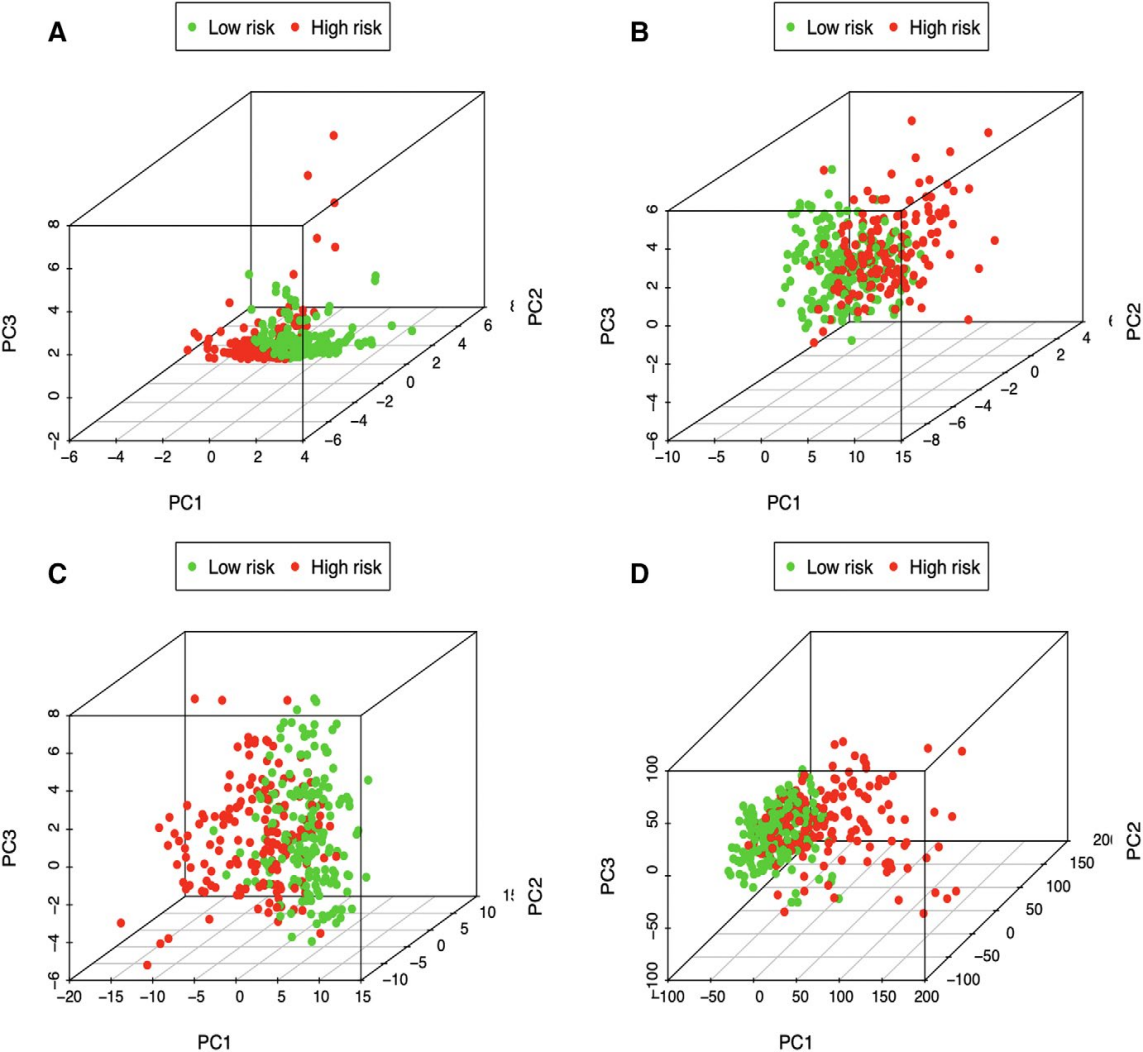
Principal components analysis between low‐ and high‐risk groups based on (A) lipid metabolism‐related signature, (B) differently expressed lipid metabolic genes, (C) all genes related to lipid metabolism, and (D) the entire gene expression profiles

We further performed principal component analysis (PCA) for examining the heterogeneity in high‐risk group compared with low‐risk group according to lipid metabolism‐associated signature (Figure 7A), differently expressed lipid metabolism‐related genes (Figure 7B), all genes related to lipid metabolism (Figure 7C), and the entire gene expression profiles (Figure 7D). As a result, the high‐ and low‐risk groups showed diverse distribution directions based on our model. Nonetheless, as shown in Figure 7B‐D, the scattered distributions of high‐ and low‐risk groups were observed, confirming the potency of our prognostic signature in the discriminating low‐ and high‐risk groups.

### 3.4 Correlation of the prognostic model with clinicopathological characteristics

**FIGURE 8:**
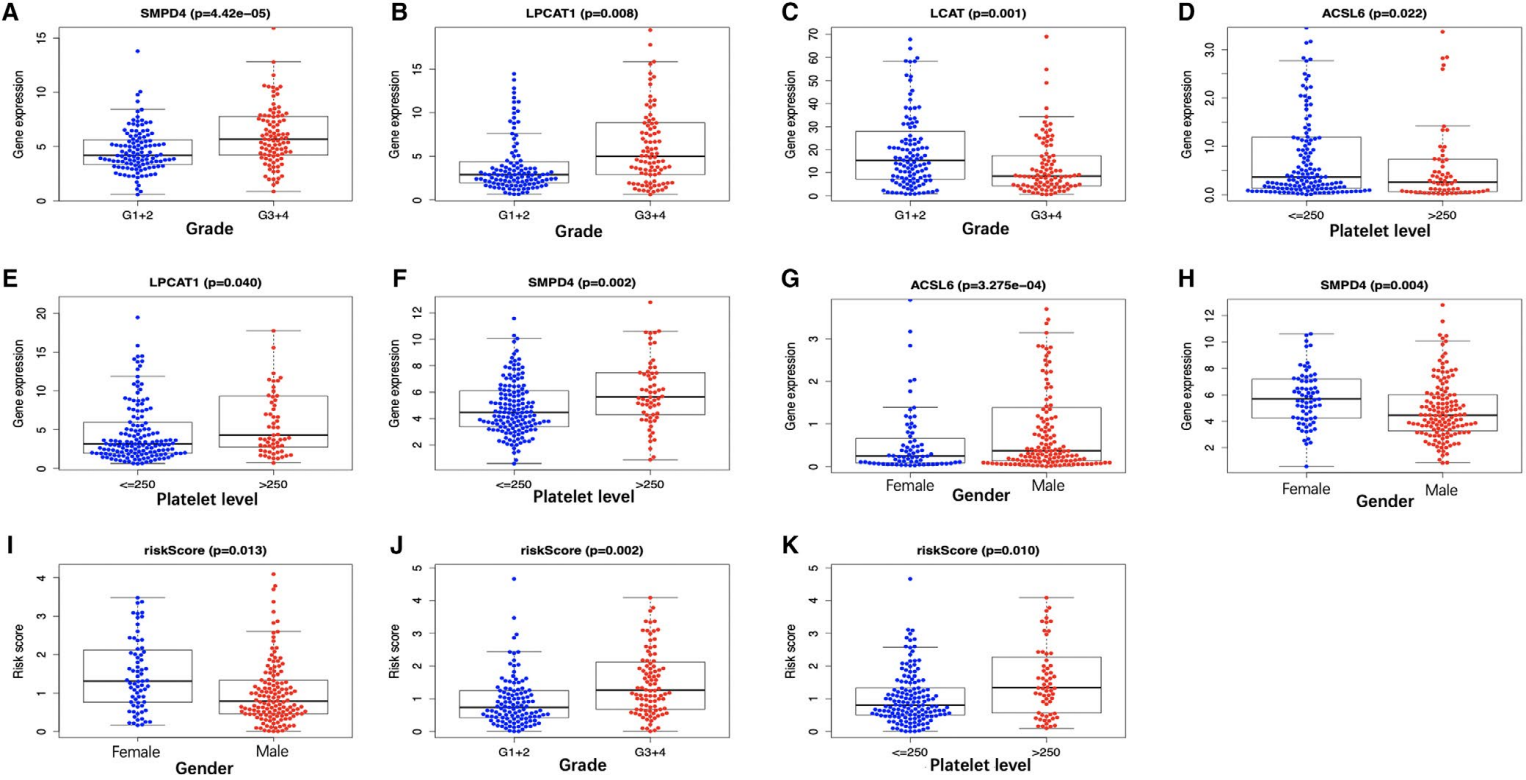
Correlation of the prognostic signature with clinicopathological characteristics based on The Cancer Genome Atlas. The expression of (A) *SMPD4*, (B) *LPCAT1*, and (C) *LCAT* is associated with tumor grade, and (D) *ACSL6,* (E) *LPCAT1*, and (F) *SMPD4* are linked with platelet level. Besides, the expression level of *ACSL6* is significantly enhanced in male patients (G), and the expression of *SMPD4* is significantly increased in female cases (H). Furthermore, the risk score was significantly correlated with patient gender (I), tumor grade (J), and platelet level (K)

A total of 216 patients with complete information, including gender, age, clinical stage, tumor grade, T stage, platelet content, albumin content, alpha‐fetoprotein (AFP) content, as well as vascular invasion, were enrolled into the TCGA HCC cohort. Across our investigated signature genes, *SMPD4*, *LPCAT1*, and *LCAT* were correlated with tumor grade (Figure 8A‐C), and *ACSL6, LPCAT1* and *SMPD4* were associated with platelet level (Figure 8D‐F). In addition, *ACSL6* level (Figure 8G) apparently elevated among male cases, while female cases were more likely to harbor high expression of *SMPD4* (Figure 8H). Furthermore, the risk score showed significant correlation with the gender, platelet level, and histological grade of patients (Figure 8I‐K; *P* < .05).

### 3.5 Gene set variation analysis validated different lipid metabolism status in low‐ or high‐risk group

**FIGURE 9:**
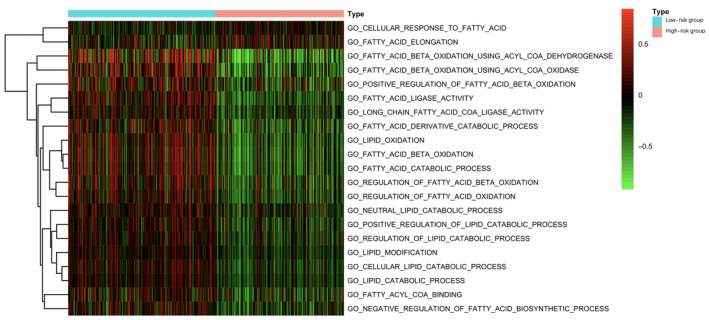
Gene set variation analysis reveals the difference in lipid metabolism between high‐ and low‐risk patients in terms of the The Cancer Genome Atlas hepatocellular carcinoma cohort. Multiple Gene Oncology terms are utilized for analysis

Afterward, we conducted GSVA for investigating difference in lipid metabolism between the low‐ and high‐risk patients from the TCGA HCC cohort. As shown by our results, the expression of genes of GO terms involved in lipocatabolism—such as “Fatty acid beta oxidation,” “Lipid oxidation,” and “Regulation of lipid catabolic process”—was decreased in high‐risk group in comparison with that in low‐risk group, while GO items “Fatty acid elongation” and “Cellular response to fatty acid” were enriched in high‐risk group (Figure 9; Table S2). Furthermore, we also analyzed the enrichment of the as‐mentioned GO terms between the HCC samples derived from TCGA and normal hepatic samples. Based on such findings, the FA elongation function of samples in the high‐risk group was enhanced compared with normal samples, and the functions related to lipid catabolism and oxidation were reduced compared with normal samples (Table S3). On the other hand, there was no significant difference in FA elongation function between the low‐risk group samples and normal samples, while the remaining functions were significantly lower than normal tissues (Table S4).

### 3.6 The different immune infiltrating levels in low‐ compared with high‐risk TCGA‐derived HCC cases

**FIGURE 10:**
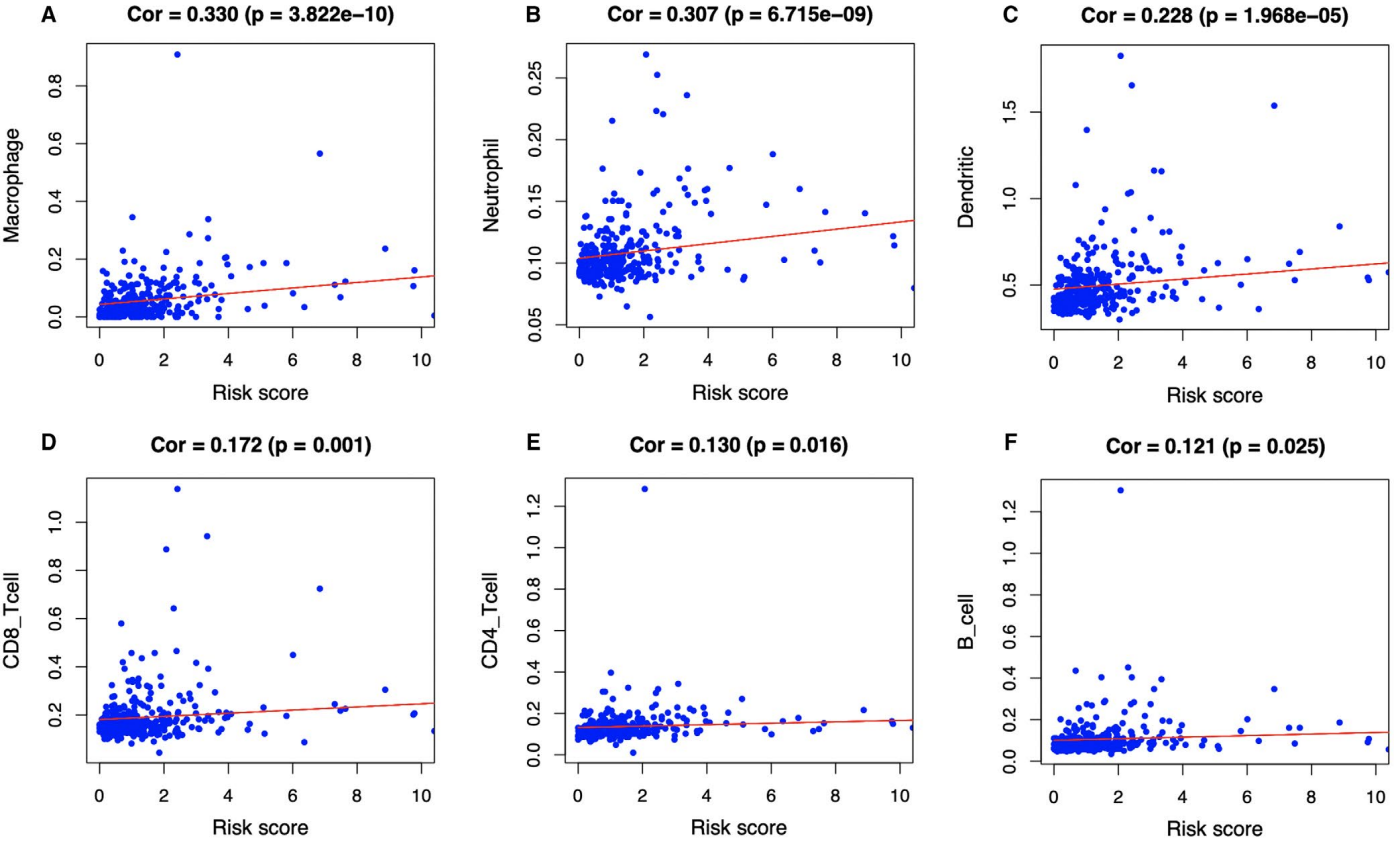
Relationships between the lipid metabolism‐related prognostic model and infiltration abundances of six types of immune cells. The correlation was performed by using Pearson correlation analysis. A, macrophages; (B) neutrophils; (C) dendritic cells; (D) CD8 + T cells; (E) CD4 + T cells; and (F) B cells

The relationship between the prognostic signature related to lipid metabolism and the infiltrating level of immunocytes among TCGA HCC cases was estimated, for the sake of examining the potential of risk score to reflect TME status. As a result, neutrophil (Cor = 0.307; *P* = 6.715e‐09), macrophage (Cor = 0.330; *P* = 3.822e‐10), as well as DC (Cor = 0.228; *P* = 1.968e‐05) levels were significantly increased in TME among high‐risk patients in the entire set (Figure 10A‐C), indicating the different immune status between high‐ and low‐risk groups. Additionally, CD8^+^ T cells (Cor = 0.172; *P* = .001) (Figure 10D), CD4^+^ T cells (Cor = 0.130; *P* = .016) (Figure 10E) as well as B cells (Cor = 0.121; *P* = .025) (Figure 10F) showed correlation with high‐risk patients.

**FIGURE 11:**
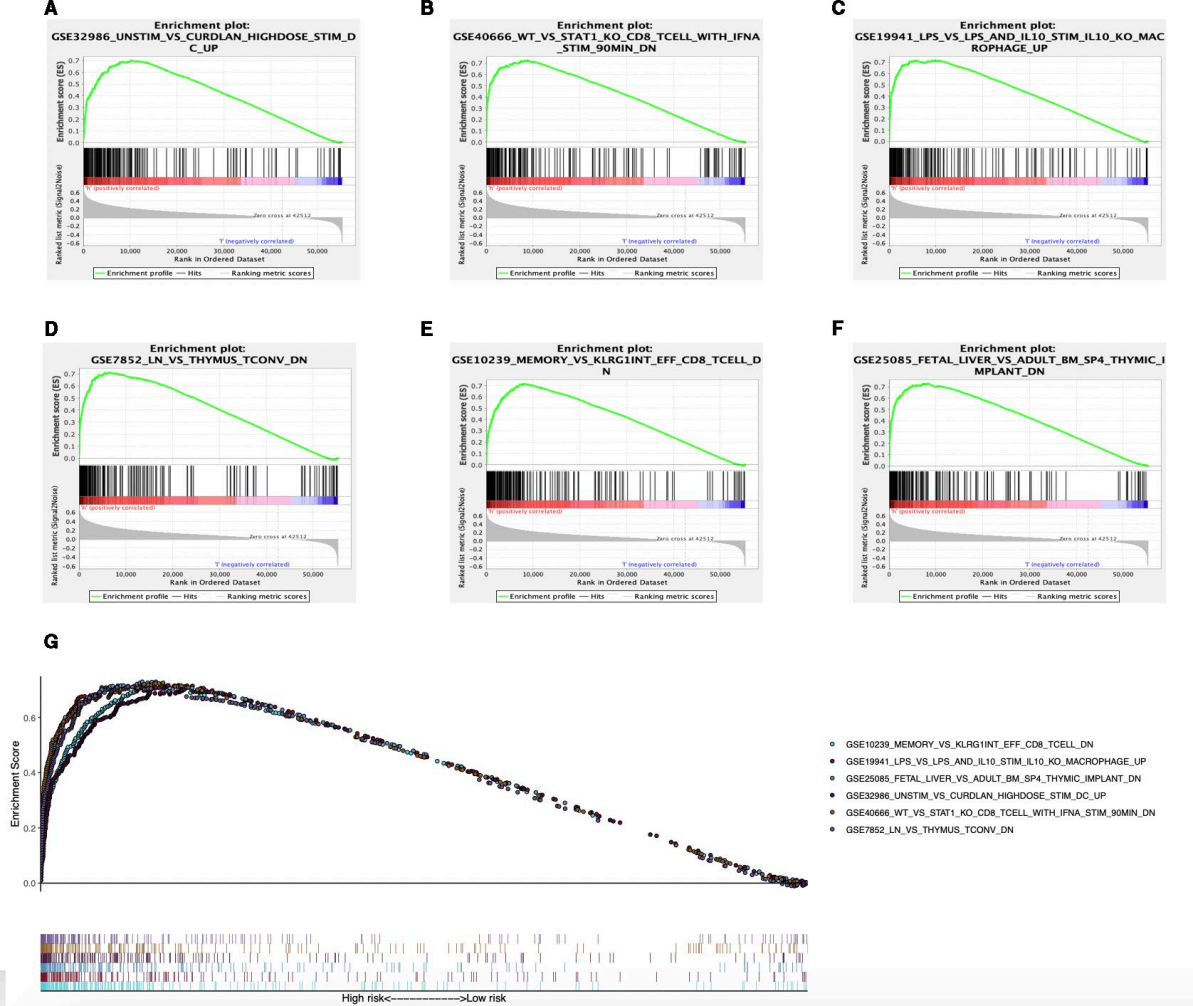
Enrichment plots of immune‐related gene sets from gene set enrichment analysis (GSEA). GSEA results showing gene sets in (A) GSE32986, (B) GSE40666, (C) GSE19941, (D) GSE7852, (E) GSE10239, and (F) GSE25085 are differentially enriched in high‐risk phenotype. G, Summarizes the above six gene sets

**FIGURE 12:**
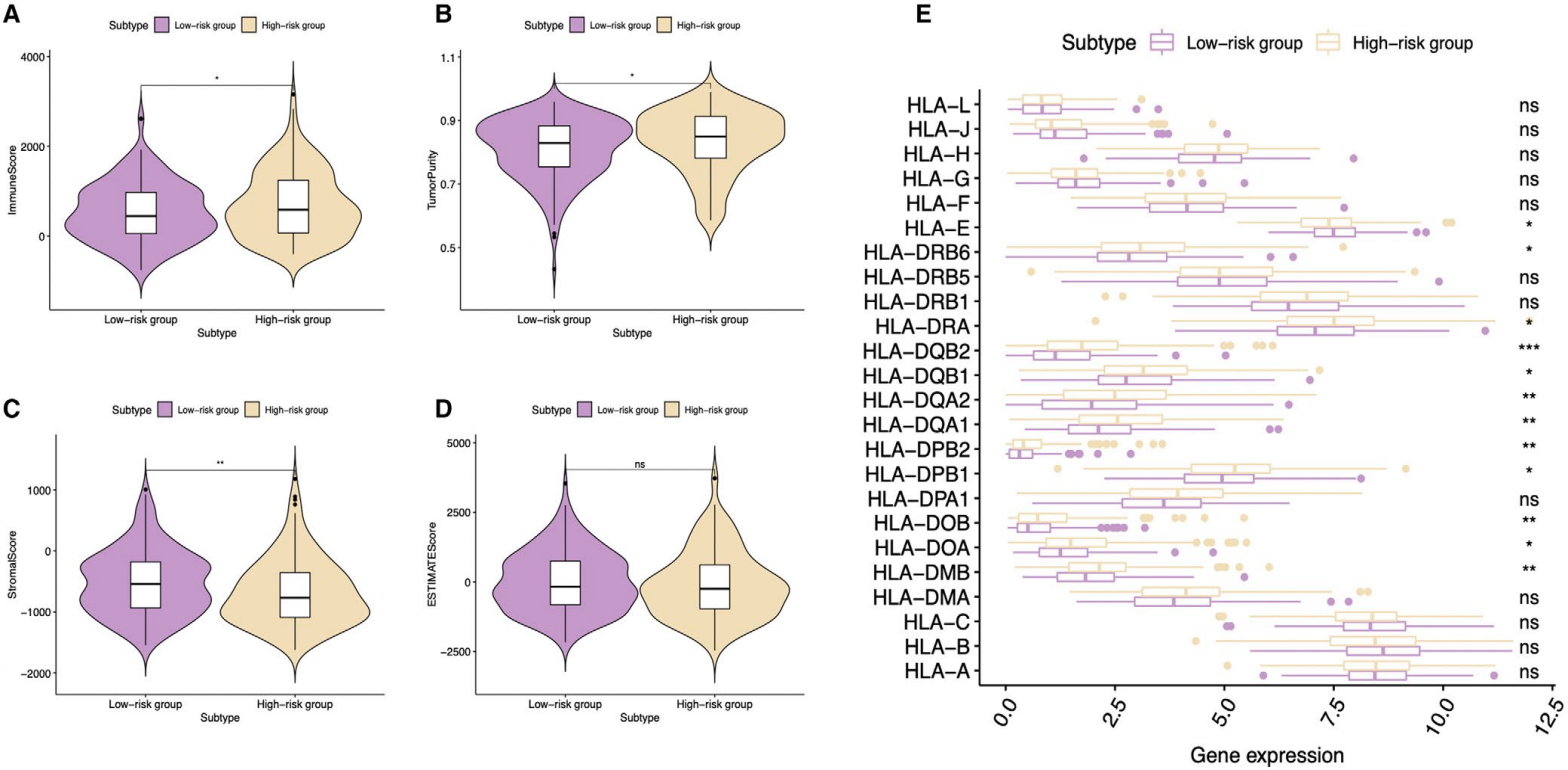
Analysis of different immune status in high‐ and low‐risk groups of The Cancer Genome Atlas hepatocellular carcinoma cohort. A, Comparison of (A) immune score, (B) tumor purity, (C) stromal score, and (D) ESTIMATE score between high‐ and low‐risk groups is shown. E, Comparison of the expression levels of human leukocyte antigen (HLA) genes between high‐ and low‐risk groups

Moreover, we conducted GSEA for investigating immune status between high‐ and low‐risk groups, showing that the DEGs were mainly enriched into several gene sets of the immunological signature (c7. All. V7.0. symbol). Besides, the six immune‐associated gene sets with the highest NES values were presented in Figure 11A‐F and Table S5. Figure 11G exhibited the comprehensive diagrams showing the above items. Subsequently, the immune score and tumor purity of high‐risk group were significantly higher than those of low‐risk group, while the stromal score was lower than that of the low‐risk group (Figure 12A‐C). However, there was no significant difference in the ESTIMATE score between the two groups (Figure 12D). It is worth noting that several human leukocyte antigen (HLA) genes achieved significantly higher expression levels in high‐risk group than those in low‐risk group (*P* < .05) (Figure 12E).

**FIGURE 13:**
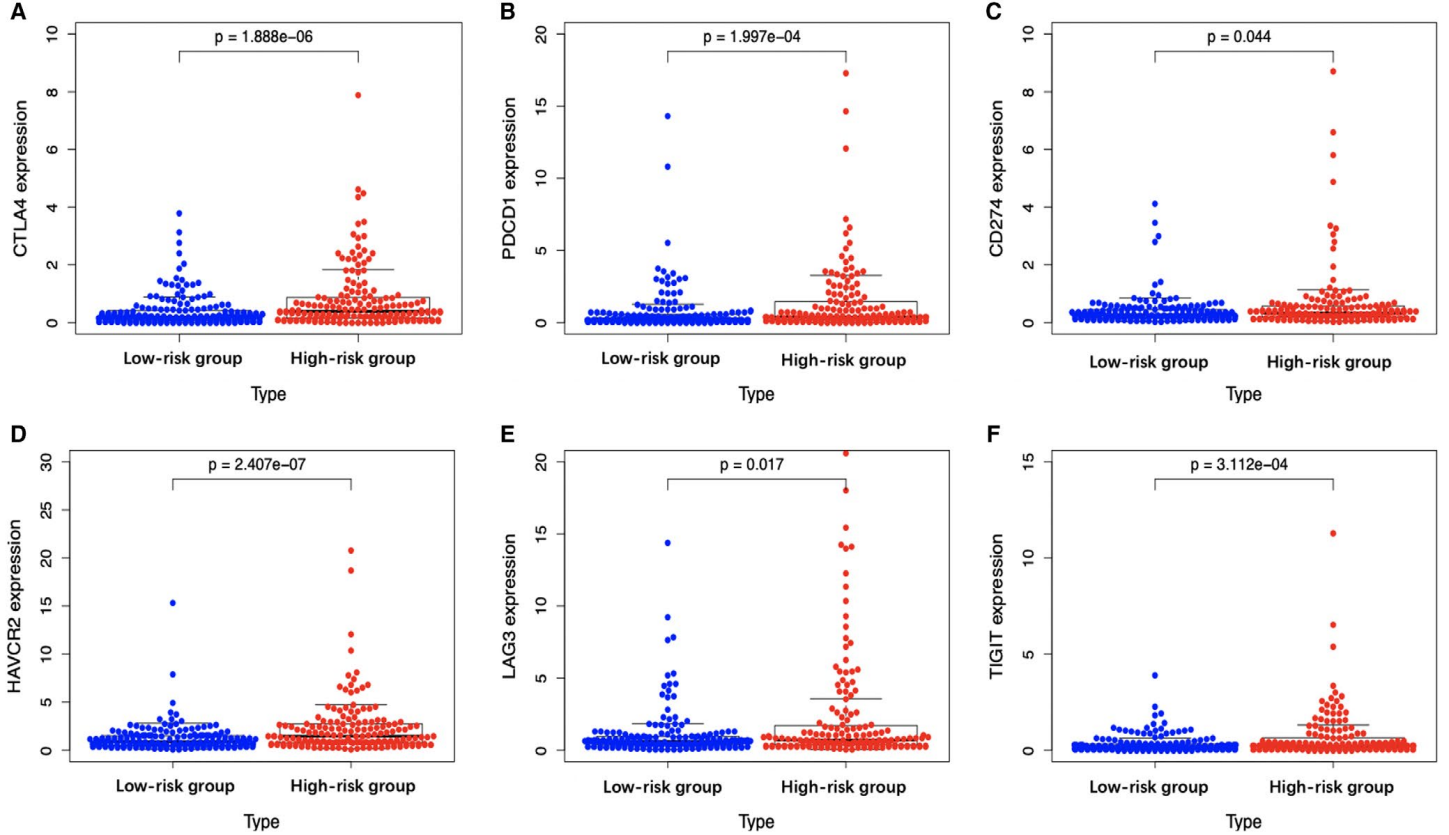
Scatter plots visualizing significantly different immune checkpoints between high‐risk and low‐risk cases. CTLA‐4, cytotoxic T‐lymphocyte associated protein 4; LAG3, lymphocyte activation gene‐3; PD‐1, programmed cell death 1; TIGIT, T‐cell immunoreceptor with Ig and ITIM domains

In consideration that immunotherapy has become an established pillar of anticancer treatment with improved prognosis among a large number of patients with various types of malignancies, we further explored the expression of common immune checkpoints and genes associated with T cell exhaustion in the high‐ and low‐risk HCC patients. The result was indicative of a significantly higher levels for programmed death ligand 1 (PD‐L1), program death 1 (PD‐1), cytotoxic T‐lymphocyte‐associated protein 4 (CTLA‐4), T‐cell immunoreceptor with Ig and ITIM domains (TIGIT), lymphocyte activation gene‐3 (LAG3), and T‐cell immunoglobulin and mucin‐domain containing‐3 (TIM‐3) of high‐risk group relative to low‐risk group (*P* < .05) (Figure 13A‐F). These above outcomes suggested that the aberrant lipid metabolism could shape the immunosuppressive microenvironment and TME of high‐risk group, which might account for the relatively dismal prognosis for these patients.

### 3.7 The qRT‐PCR results

**FIGURE 14:**
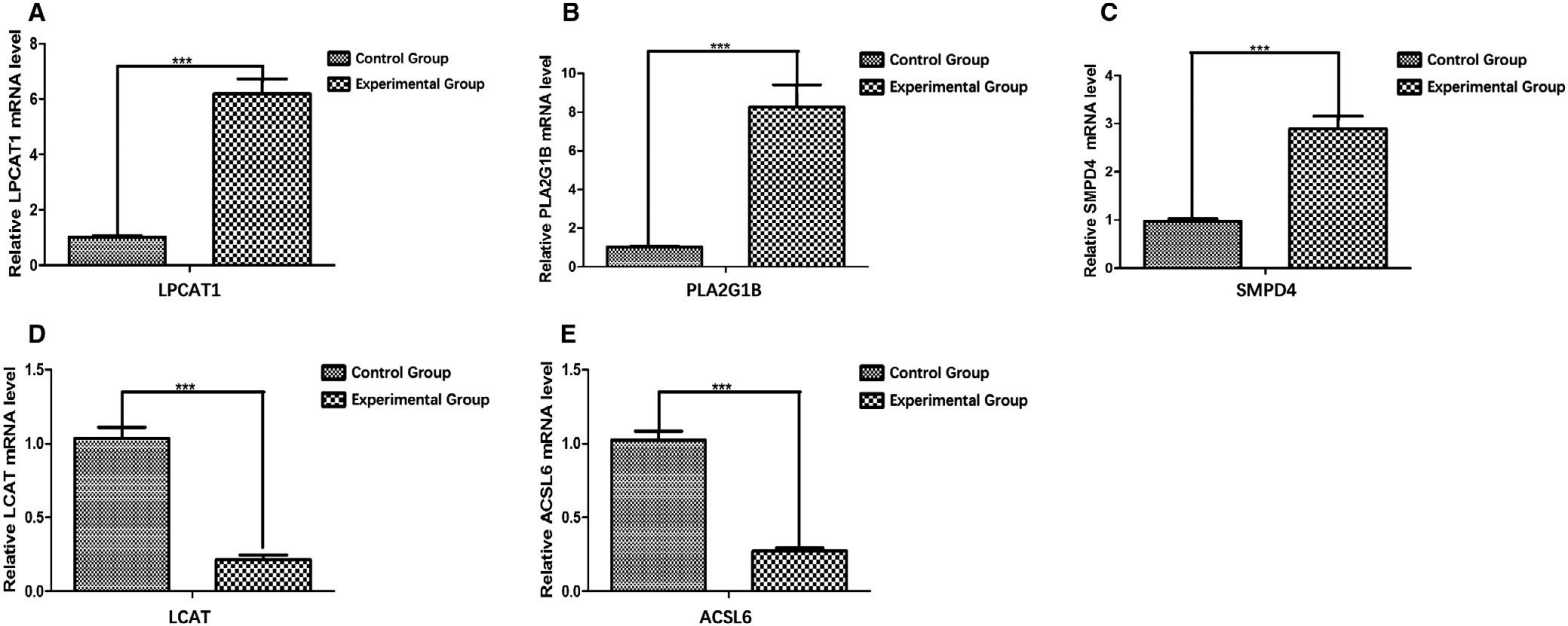
The quantitative real‐time polymerase chain reaction (qRT‐PCR) validation of five genes included in the prognostic model. A, *LPCAT1*, (B) *PLA2G1B*, and (C) *SMPD4* were significantly overexpressed in the experimental group (hepatocellular carcinoma tissues) compared with the control group (para‐carcinoma tissues). The expression of (D) *LCAT* and (E) *ACSL6* was significantly lower in the experimental group (hepatocellular carcinoma tissues) compared with the control group (para‐carcinoma tissues). *P < .05; **P < .01; ***P < .001

Subsequently, the expression level of five signature genes (*LPCAT1, ACSL6, PLA2G1B, LCAT,* and *SMPD4*) was validated by qRT‐PCR. As a result, *LPCAT1, PLA2G1B,* and *SMPD4* were significantly overexpressed in the experimental group (HCC tissues) compared with the control group (para‐carcinoma tissues) (*P* < .001) (Figure 14A‐C), while the expression of *LCAT* and *ACSL6* were significantly lower in the experimental group (HCC tissues) compared with the control group (para‐carcinoma tissues) (*P* < .001) (Figure 14D‐E), which is consistent with our bioinformatics analysis. The expression of five genes in each tissue is exhibited in Figure S3. The primer sequences are shown in Table S6.

## 4 DISCUSSION

Accumulative evidence has suggested that alterations in tumor lipid metabolism could lead to tumor progression and local immunosuppression in the TME.^21^ Due to the limited efficacy of diverse therapeutic methods of HCC, like chemotherapy, radiofrequency ablation, liver transplantation, and surgical resection exploring novel biomarkers is urgently needed, which could not only predict the OS of HCC patients, but also be utilized to guide anticancer therapy. Consequently, recent studies have shed more light on the aberrant lipid metabolism and its effect on the immune microenvironment in the context of HCC.

In the present work, we carried out complicated bioinformatic analyses to successfully identify five lipid metabolism‐related genes that were associated with HCC prognosis, including *ACSL6, LPCAT1, PLA2G1B, LCAT,* and *SMPD4*. To begin with, we first investigated those heterogeneities in lipid metabolism‐related gene expression patterns in HCC tissue samples compared with noncarcinoma tissue samples, and subsequently identified prognosis‐related genes. Afterward, a TF‐mediated network was constructed, aiming to identify critical TFs and to reveal the precise molecular mechanisms. Typically, the chromobox protein homolog 3 had most nodes associated with the survival‐associated metabolic genes, and it is demonstrated to enhance cancer proliferation and be capable of predicting poor survival in HCC.^22^ Another impressive factor, euchromatin histone methyltransferase 2, has also been illustrated to be involved in the metastasis and the invasion of HCC.^23^ In addition, heat shock factor 2, revealed by our network and essential for survival among all organisms under acute stress, has been reported to indicate unfavorable prognosis of HCC patients and regulate aerobic glycolysis by suppressing fructose‐bisphosphatase 1 to support the uncontrolled proliferation of HCC cells.^24^ To sum up, this study has presented the integrative lipid metabolic gene regulatory network related to HCC. Nevertheless, more researches should be conducted for examining the more connections of these genes with their influences on HCC.

Afterward, a lipid metabolism‐related risk score model based on the as‐mentioned five genes was constructed and shown. Notably, it can discriminate high‐risk from low‐risk population, and the prognostic estimation is highly accurate. HCC patients in low‐risk group were proved to have longer OS than those in high‐risk group in three TCGA HCC sets and ICGC HCC cohort. Additionally, our constructed prognostic signature in this study can potentially estimate the different prognosis for low‐ and high‐risk groups based on stratification of clinical stage (I and II/III and IV), age (≤60/>60), T stage (T1 and T2/T3 and T4), and grade (G1 and G2/G3 and G4). In terms of the clinical utility, the prognostic model was significantly correlated with the gender, platelet level, and tumor grade of HCC patients from TCGA data set, indicating markedly increased risk score determined using our constructed model in female patients and patients with higher platelet level and advanced grade. Despite the scarce investigations on the relationship between platelet levels and lipid metabolism reprogramming and the development of HCC, the role of platelet‐derived growth factor‐C in liver fibrosis occurrence, including collagen deposition in the perivenular and pericellular manner, cell steatosis, and HCC progression, has been elucidated.^25^ The ratio of mean platelet volume to platelet count is also examined within liver disorders such as HCC, cirrhosis, and steatosis.^26^, ^27^ Further PCA confirmed that our model showed sound stratification ability, and the expression level of the five signature genes is also verified by qRT‐PCR. Consequently, the lipid metabolism‐related signature identified may be involved in the occurrence and development of HCC, rendering its potential as the valuable clinical biomarker.

Furthermore, we examined the difference in lipid metabolism between low‐ and high‐risk groups via GSVA. As a result, patients in high‐risk group showed a lower FA oxidation (FAO) and a higher FA elongation than those in the low‐risk group. Decreased FAO has been reported in various HCC cases,^28^, ^29^ providing robust support for our results. Recently, Gholamreza et al have discovered three subtypes of HCC, which were called iHCC1‐3, of them, iHCC1 has the greatest FAO fluxes and survival rate, which may be associated with the higher inflammatory and immune responses.^30^ On the contrary, tumors of iHCC3 displayed downregulated FAO and lipid oxidation, with upregulation of multiple processes related to DNA replication, cell proliferation, mitosis as well as cell cycle progression. Therefore, we speculate that relatively lower FAO in HCC patients may predict worse prognosis, which is consistent with our analysis. On the other hand, elongation of FAs has also been elaborated to promote hepatic lipid accumulation and participate in the progression of nonalcoholic steatohepatitis (NASH)‐related HCC.^31^ Another GO term enriched in high‐risk group, “cellular response to fatty acid”, which can be interpreted into any process stimulated by FA and resulted in cellular change or activity, also reflected that patients in high‐risk group are more likely to be affected by lipid accumulation.

Subsequently, this study suggested that, our signature was positively related to infiltrating levels for six types of immunocytes, in particular for neutrophils and macrophages, which indicated the presence of high infiltrating levels for such cells among high‐risk cases. Notably, intracellular lipids are cumulated in several tumor‐associated macrophages (TAMs), which suggests that they are metabolically active and exert immunomodulatory effects. The macrophage lipid loading is reported to be related to the elevating inflammatory and antitumor capacities.^32^, ^33^ Moreover, the increased infiltration of TAM, possibly as a result of the Hippo signaling deletion, is suggested to activate the Wnt/β‐catenin signal transduction pathway, which in turn accelerates HCC progression.^34^, ^35^ On the other side, the CXCL5 and CXCR2‐CXCL1 axes have been elaborated to promote intramural neutrophil infiltration, which is related to the shorter OS and HCC recurrence.^36^, ^37^ Meanwhile, according to Lodhi et al, peroxisomal lipid synthesis drives inflammation through promoting neutrophil membrane viability and phospholipid composition.^38^ Nonetheless, rare studies have examined the role of aberrant lipid metabolism in neutrophil proliferation and functions in HCC. Moreover, several HLA genes, such as HLA‐DRA, HLA‐DQB1, and HLA‐DQB2, were shown to be overexpressed in high‐risk group. Matoba et al have reported that tumor HLA‐DR protein expression was one of the independent risk factors for early intrahepatic recurrence in HCC patients.^39^ Besides, rs9275319 at HLA‐DQ was deemed as an independent loci that were significantly associated with HCC development.^40^ However, there are still few reports concerning on the effect and mechanism of abnormal lipid metabolism on HLA genes and the effect of the combination of both on HCC progression, and this is also one of our future research contents.

Furthermore, the expression levels of immune checkpoints in low‐ and high‐risk HCC cases were also measured. As a result, PD‐1, PD‐L1, CTLA‐4, TIM‐3, and LAG3 expression significantly increased among high‐risk HCC patients relative to low‐risk counterparts (*P* < .05). Dorn et al have reported that, the PD‐L1 level is upregulated within primary human hepatocytes using the hepatocellular lipid accumulation model in vitro, while it is confirmed through human liver specimen analysis that, PD‐1 and PD‐L1 levels are upregulated among NASH cases, which emphasizes the potential effect of aberrant lipid metabolism on immune checkpoint expression.^41^ More importantly, such findings indicate that, immune checkpoint antibody treatments, such as nivolumab (anti‐PD1 antibody) and ipilimumab (anti‐CTLA‐4 antibody),^42^ will bring more therapeutic benefit to the high‐risk HCC cases than to low‐risk counterparts, thus leading to superior outcomes. As for the treatments of high‐risk patients from lipid metabolic reprogramming, fibronectin type III domain‐containing 5 has been illustrated to increase FAO and autophagy of hepatocytes through AMPK/mTORC1 signaling pathway, to reduce de novo synthesis of lipid, thereby alleviating damage.^43^ Another agent, metformin, which suppresses the cholesterol and lipid, as well as hepatocellular glucose biosynthesis pathways through the transcriptional suppression of the steroid receptor coactivator 2 and increase of FAO, may also benefit the high‐risk patients.^44^ However, considering the tumor biological and genotypic diversities among HCC cases, together with the complicated lipid metabolism, more efforts should be made to investigate the treatment strategies that target the FA‐related pathways for the treatment of HCC.

However, some limitations should be noted in this study. The present study mainly focused on bioinformatics analysis. Although the expression level of the five model genes in HCC and para‐carcinoma tissues was identified by qRT‐PCR, this study still lacks mechanism verification. Our consequent work will continue to focus on the specific mechanisms of these genes in HCC.

## 5 CONCLUSION

To sum up, this work established and validated the prognostic signature on the basis of five lipid metabolism‐associated genes, for the sake of predicting HCC OS. This prognostic signature can help to select the individualized therapeutic strategy in clinical practice. In addition, our risk score model could connect with lipid metabolism and immune status, which provides a comprehensive perspective for clarifying the underlying mechanisms that determine the prognosis for HCC.

## CONFLICT OF INTEREST

The authors declare that they have no competing interests.

## AUTHOR CONTRIBUTIONS

Xin‐Ting Sang and Bo Hu created the idea for the paper. Bo Hu performed the collection and assembly of data, conducted the analysis, drafted the manuscript, and prepared the figures. Xiao‐Bo Yang and Xin‐Ting Sang revised the manuscript. All authors read and approved the final manuscript.

## Supporting information

FigS1

FigS2

FigS3

Table S1

Table S2

Table S3

Table S4

Table S5

Table S6

Supplementary Materials

## Data Availability

All data generated or analyzed during this study are included in this article and its Supporting Information files.
