# Supplementary figures and images for: Construction of a lipid metabolism‐related and immune‐associated prognostic signature for hepatocellular carcinoma
Source: Cancer Med. 2020 Aug 19;9(20):7646–62. doi: 10.1002/cam4.3353 (PMC7571839; doi:10.1002/cam4.3353)

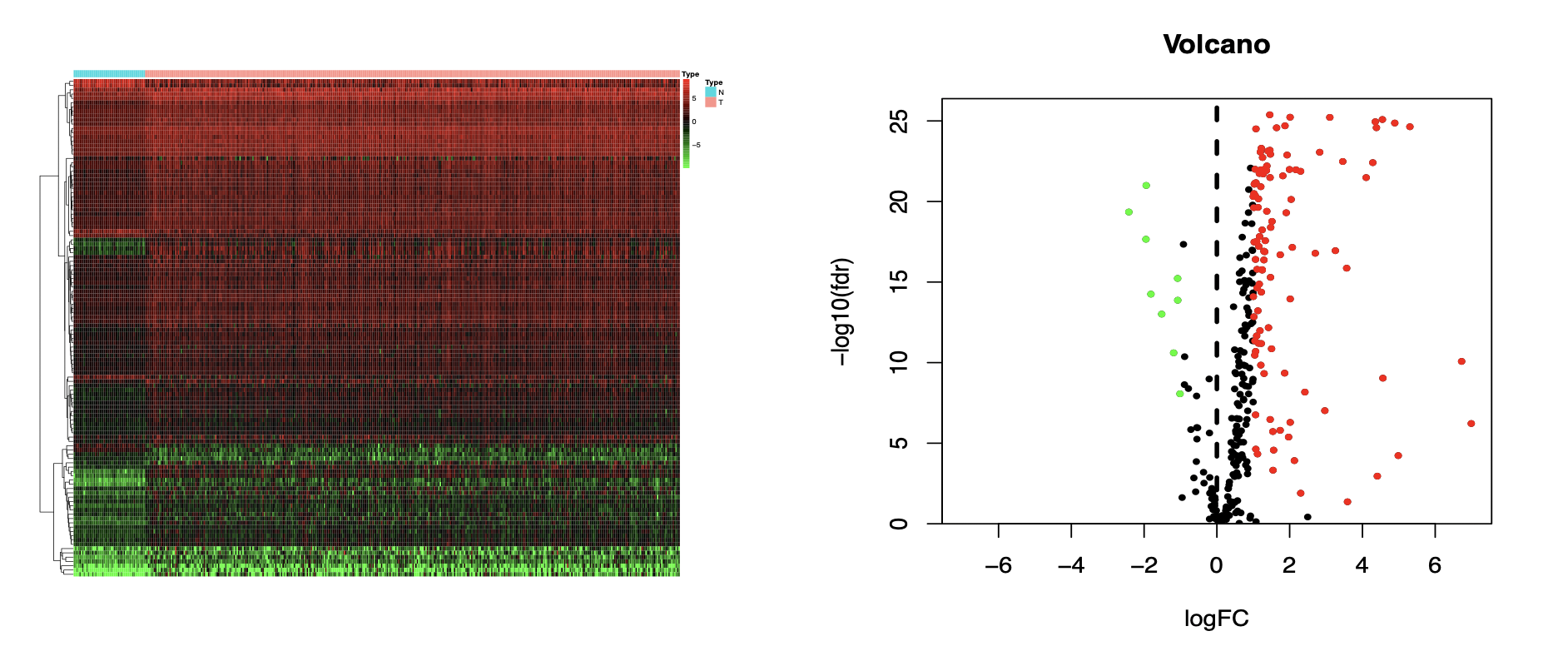

Supplement: Supplementary file 1 — FigS1 [file CAM4-9-7646-s001.tiff]

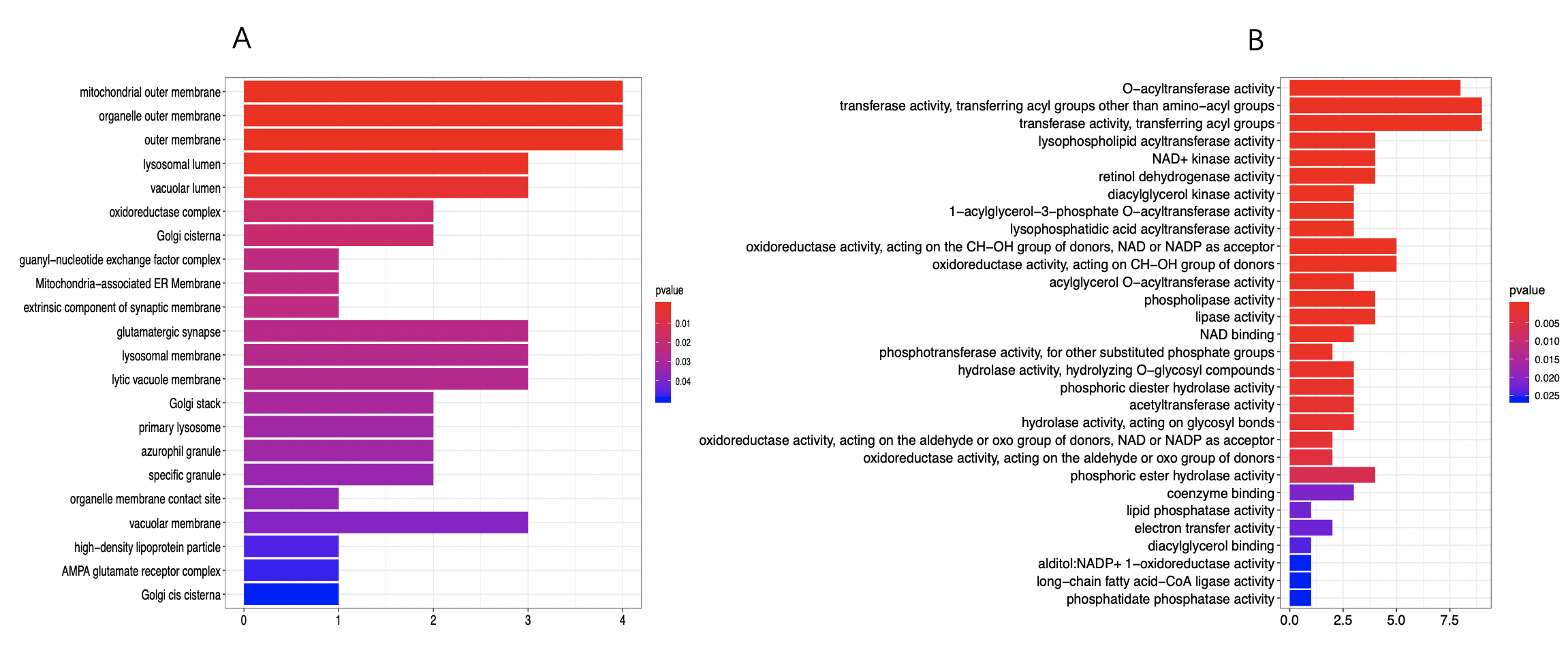

Supplement: Supplementary file 2 — FigS2 [file CAM4-9-7646-s002.tiff]

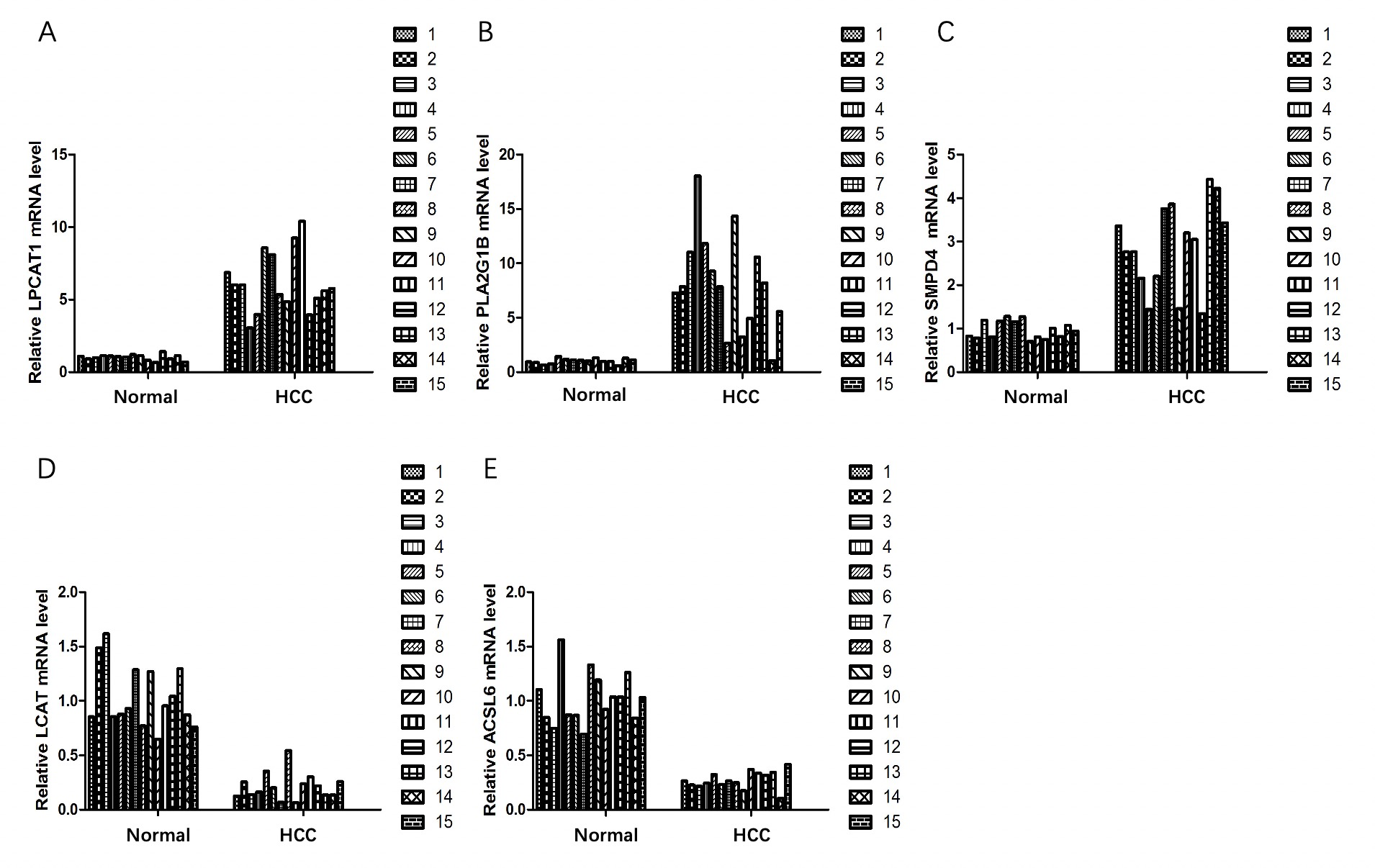

Supplement: Supplementary file 3 — FigS3 [file CAM4-9-7646-s003.tiff]
