# Supplementary material for: Construction of a lipid metabolism‐related and immune‐associated prognostic signature for hepatocellular carcinoma
Source: Cancer Med. 2020 Aug 19;9(20):7646–62. doi: 10.1002/cam4.3353 (PMC7571839; doi:10.1002/cam4.3353)
Supplement: Supplementary file 4 — Table S1 [file CAM4-9-7646-s004.docx]

Supplementary Table 1. General characteristics of TCGA HCC-specific survival-related lipid metabolism-related genes

| id | HR | HR.95L | HR.95H | P-value |
| --- | --- | --- | --- | --- |
| LPCAT1 | 1.05943479 | 1.03657655 | 1.0827971 | 2.13E-07 |
| MBOAT7 | 1.09031295 | 1.05141424 | 1.13065079 | 3.09E-06 |
| SMPD4 | 1.21228269 | 1.11289454 | 1.3205468 | 1.03E-05 |
| GPD2 | 1.48314615 | 1.24423456 | 1.76793234 | 1.09E-05 |
| PTDSS2 | 1.14652994 | 1.07826175 | 1.21912039 | 1.27E-05 |
| PGS1 | 1.23451582 | 1.09183001 | 1.39584852 | 0.00077403 |
| GPD1L | 1.2440179 | 1.0906549 | 1.41894611 | 0.00114315 |
| PLA2G1B | 1.05932732 | 1.02252157 | 1.09745788 | 0.00140134 |
| DGKZ | 1.17952708 | 1.06233081 | 1.30965243 | 0.00198529 |
| DGKD | 1.35008424 | 1.11225456 | 1.63876825 | 0.00239712 |
| LPCAT4 | 1.15445569 | 1.05181321 | 1.26711466 | 0.00250054 |
| GLA | 1.02953012 | 1.01023045 | 1.0491985 | 0.00257701 |
| ADH1A | 0.99662115 | 0.99433706 | 0.99891048 | 0.00383832 |
| DGKI | 3.96511536 | 1.52597006 | 10.3030461 | 0.00469244 |
| LCAT | 0.96792873 | 0.94546825 | 0.99092277 | 0.00650466 |
| ACSL6 | 0.57250068 | 0.38182275 | 0.858401 | 0.00695983 |
| SMPD2 | 1.19157774 | 1.04867318 | 1.35395616 | 0.00716454 |
| ADH1B | 0.99782956 | 0.99624137 | 0.99942028 | 0.00750709 |
| ALDH2 | 0.9902489 | 0.98292825 | 0.99762407 | 0.00964544 |
| AGPAT1 | 1.03563858 | 1.00852417 | 1.06348197 | 0.00968063 |
| SMPD3 | 1.25089753 | 1.05250655 | 1.486684 | 0.01106038 |
| CPT1C | 1.19855586 | 1.0378411 | 1.3841581 | 0.01367852 |
| ADH1C | 0.99839191 | 0.99710968 | 0.99967579 | 0.01410768 |
| DEGS1 | 1.02824225 | 1.00555105 | 1.05144549 | 0.01443823 |
| CHKA | 1.02972955 | 1.00540548 | 1.05464209 | 0.01630751 |
| ADH4 | 0.99859879 | 0.99740847 | 0.99979053 | 0.0212106 |
| CYP4A11 | 0.99405504 | 0.98897261 | 0.99916359 | 0.02261301 |
| PLPP1 | 0.98102244 | 0.96488397 | 0.99743084 | 0.02357873 |
| GPAT2 | 1.14381131 | 1.01578338 | 1.28797569 | 0.02651873 |
| GBA | 1.02240097 | 1.00247797 | 1.04271992 | 0.02735194 |
| SPHK1 | 1.02318864 | 1.00248705 | 1.04431771 | 0.02793849 |
| CHKB | 1.36182994 | 1.03122723 | 1.79842107 | 0.02950345 |
| NEU1 | 1.01310138 | 1.00121445 | 1.02512944 | 0.03065548 |
| B4GALT6 | 1.14362368 | 1.01187462 | 1.29252686 | 0.03163489 |
| GAL3ST1 | 1.01541658 | 1.00067725 | 1.030373 | 0.04029476 |
| AGPAT4 | 1.25777774 | 1.00757808 | 1.57010645 | 0.04269544 |
