# Supplementary material for: Construction of a lipid metabolism‐related and immune‐associated prognostic signature for hepatocellular carcinoma
Source: Cancer Med. 2020 Aug 19;9(20):7646–62. doi: 10.1002/cam4.3353 (PMC7571839; doi:10.1002/cam4.3353)
Supplement: Supplementary file 5 — Table S2 [file CAM4-9-7646-s005.docx]

| id | logFC | AveExpr | t | P.Value | adj.P.Val |
| --- | --- | --- | --- | --- | --- |
| GO_CELLULAR_LIPID_CATABOLIC_PROCESS | -0.2864732 | -0.0512657 | -12.508596 | 6.54E-30 | 8.04E-28 |
| GO_CELLULAR_RESPONSE_TO_FATTY_ACID | 0.11037159 | -0.0556593 | 5.13867599 | 4.62E-07 | 3.05E-05 |
| GO_FATTY_ACID_BETA_OXIDATION | -0.4286441 | -0.0718266 | -13.05839 | 5.31E-32 | 6.74E-30 |
| GO_FATTY_ACID_BETA_OXIDATION_USING_ACYL_COA_DEHYDROGENASE | -0.5396337 | -0.1098876 | -11.770176 | 3.71E-27 | 4.46E-25 |
| GO_FATTY_ACID_BETA_OXIDATION_USING_ACYL_COA_OXIDASE | -0.463916 | -0.0680064 | -12.465969 | 9.47E-30 | 1.16E-27 |
| GO_FATTY_ACID_CATABOLIC_PROCESS | -0.4101301 | -0.0706231 | -12.989556 | 9.73E-32 | 1.22E-29 |
| GO_FATTY_ACID_DERIVATIVE_CATABOLIC_PROCESS | -0.3388317 | -0.0810941 | -10.161474 | 2.03E-21 | 2.27E-19 |
| GO_FATTY_ACID_ELONGATION | 0.12755002 | -0.0174652 | 4.14765487 | 4.22E-05 | 2.53E-03 |
| GO_FATTY_ACID_LIGASE_ACTIVITY | -0.4012533 | -0.0671886 | -14.324941 | 6.36E-37 | 8.26E-35 |
| GO_FATTY_ACYL_COA_BINDING | -0.2160285 | -0.0777563 | -7.0472163 | 9.82E-12 | 8.05E-10 |
| GO_LIPID_CATABOLIC_PROCESS | -0.2402738 | -0.0522043 | -12.229324 | 7.33E-29 | 8.86E-27 |
| GO_LIPID_MODIFICATION | -0.2170855 | -0.0535154 | -11.311025 | 1.77E-25 | 2.10E-23 |
| GO_LIPID_OXIDATION | -0.3979652 | -0.0722684 | -13.026648 | 7.02E-32 | 8.85E-30 |
| GO_LONG_CHAIN_FATTY_ACID_COA_LIGASE_ACTIVITY | -0.2874895 | -0.0566587 | -10.992976 | 2.46E-24 | 2.88E-22 |
| GO_NEGATIVE_REGULATION_OF_FATTY_ACID_BIOSYNTHETIC_PROCESS | -0.2355113 | -0.0741361 | -8.2020655 | 4.59E-15 | 4.46E-13 |
| GO_NEUTRAL_LIPID_CATABOLIC_PROCESS | -0.2126759 | -0.0176657 | -8.8276676 | 5.27E-17 | 5.37E-15 |
| GO_POSITIVE_REGULATION_OF_FATTY_ACID_BETA_OXIDATION | -0.2895692 | -0.0448009 | -8.114174 | 8.46E-15 | 8.04E-13 |
| GO_POSITIVE_REGULATION_OF_LIPID_CATABOLIC_PROCESS | -0.2419416 | -0.0472169 | -9.2003012 | 3.35E-18 | 3.48E-16 |
| GO_REGULATION_OF_FATTY_ACID_BETA_OXIDATION | -0.3205035 | -0.0584228 | -9.7044727 | 7.23E-20 | 7.81E-18 |
| GO_REGULATION_OF_FATTY_ACID_OXIDATION | -0.317621 | -0.0705469 | -11.203641 | 4.32E-25 | 5.09E-23 |
| GO_REGULATION_OF_LIPID_CATABOLIC_PROCESS | -0.2244514 | -0.0526029 | -9.3886413 | 8.10E-19 | 8.59E-17 |

Supplementary Table 2. General characteristics of the differently enriched Gene Oncology terms between high- and low-risk group with regard to TCGA

FC, fold change (high-risk group vs. low-risk group); AveExpr, average expression; adj, adjusted.
