# Supplementary material for: Construction of a lipid metabolism‐related and immune‐associated prognostic signature for hepatocellular carcinoma
Source: Cancer Med. 2020 Aug 19;9(20):7646–62. doi: 10.1002/cam4.3353 (PMC7571839; doi:10.1002/cam4.3353)
Supplement: Supplementary file 6 — Table S3 [file CAM4-9-7646-s006.docx]

| id | logFC | AveExpr | t | P.Value | adj.P.Val |
| --- | --- | --- | --- | --- | --- |
| GO_FATTY_ACID_ELONGATION | 0.16647332 | 0.0103486 | 3.76998441 | 0.00021054 | 0.00442138 |
| GO_CELLULAR_RESPONSE_TO_FATTY_ACID | -0.103038 | 0.02813188 | -3.7057453 | 0.00026761 | 0.00508468 |
| GO_NEGATIVE_REGULATION_OF_LIPID_BIOSYNTHETIC_PROCESS | -0.3020445 | -0.046014 | -11.673022 | 9.5766E-25 | 6.8952E-23 |
| GO_NEUTRAL_LIPID_CATABOLIC_PROCESS | -0.3640452 | -0.0431422 | -10.162106 | 4.3334E-20 | 2.8167E-18 |
| GO_LIPID_MODIFICATION | -0.4312573 | -0.065099 | -16.187283 | 3.6723E-39 | 3.9661E-37 |
| GO_POSITIVE_REGULATION_OF_LIPID_CATABOLIC_PROCESS | -0.4801368 | -0.0602474 | -11.966102 | 1.1484E-25 | 8.613E-24 |
| GO_REGULATION_OF_LIPID_CATABOLIC_PROCESS | -0.5145204 | -0.0479913 | -14.484308 | 1.0575E-33 | 1.0152E-31 |
| GO_LONG_CHAIN_FATTY_ACID_COA_LIGASE_ACTIVITY | -0.5282171 | -0.082369 | -14.421037 | 1.6883E-33 | 1.6039E-31 |
| GO_LIPID_CATABOLIC_PROCESS | -0.5324038 | -0.0517117 | -20.006952 | 3.8921E-51 | 4.9041E-49 |
| GO_POSITIVE_REGULATION_OF_FATTY_ACID_BETA_OXIDATION | -0.5420324 | -0.0682816 | -10.500791 | 4.0686E-21 | 2.8073E-19 |
| GO_CELLULAR_LIPID_CATABOLIC_PROCESS | -0.5575255 | -0.0693497 | -17.464666 | 3.1954E-43 | 3.7706E-41 |
| GO_REGULATION_OF_FATTY_ACID_BETA_OXIDATION | -0.6416185 | -0.0743371 | -13.528935 | 1.2278E-30 | 1.0805E-28 |
| GO_FATTY_ACID_LIGASE_ACTIVITY | -0.6562355 | -0.1226522 | -17.435475 | 3.9519E-43 | 4.6237E-41 |
| GO_FATTY_ACID_DERIVATIVE_CATABOLIC_PROCESS | -0.6773078 | -0.0981608 | -14.129824 | 1.4534E-32 | 1.3371E-30 |
| GO_REGULATION_OF_FATTY_ACID_OXIDATION | -0.6842508 | -0.0746189 | -17.223318 | 1.8547E-42 | 2.0772E-40 |
| GO_FATTY_ACID_BETA_OXIDATION_USING_ACYL_COA_OXIDASE | -0.7755588 | -0.1280611 | -15.304508 | 2.462E-36 | 2.5113E-34 |
| GO_LIPID_OXIDATION | -0.7949109 | -0.092459 | -19.977765 | 4.7808E-51 | 5.976E-49 |
| GO_FATTY_ACID_BETA_OXIDATION | -0.8238305 | -0.1013952 | -19.533695 | 1.1045E-49 | 1.3696E-47 |
| GO_FATTY_ACID_CATABOLIC_PROCESS | -0.8241373 | -0.0902398 | -20.230335 | 8.0896E-52 | 1.0274E-49 |
| GO_FATTY_ACID_BETA_OXIDATION_USING_ACYL_COA_DEHYDROGENASE | -1.1327678 | -0.1240001 | -20.461563 | 1.6003E-52 | 2.0643E-50 |

Supplementary Table 3. General characteristics of the differently enriched Gene Oncology terms between high-risk group samples and normal hepatic samples with regard to TCGA

FC, fold change (high-risk samples vs. normal samples); AveExpr, average expression; adj, adjusted.
