# Supplementary material for: Construction of a lipid metabolism‐related and immune‐associated prognostic signature for hepatocellular carcinoma
Source: Cancer Med. 2020 Aug 19;9(20):7646–62. doi: 10.1002/cam4.3353 (PMC7571839; doi:10.1002/cam4.3353)
Supplement: Supplementary file 7 — Table S4 [file CAM4-9-7646-s007.docx]

| id | logFC | AveExpr | t | P.Value | adj.P.Val |
| --- | --- | --- | --- | --- | --- |
| GO_NEUTRAL_LIPID_CATABOLIC_PROCESS | -0.1513693 | 0.11453245 | -5.1203015 | 6.2648E-07 | 2.6939E-05 |
| GO_CELLULAR_RESPONSE_TO_FATTY_ACID | -0.2134096 | -0.0606323 | -7.0260814 | 2.187E-11 | 1.4653E-09 |
| GO_LIPID_MODIFICATION | -0.2141719 | 0.09442741 | -8.5140484 | 1.8629E-15 | 1.6208E-13 |
| GO_POSITIVE_REGULATION_OF_LIPID_CATABOLIC_PROCESS | -0.2381952 | 0.11755656 | -7.5708784 | 7.9767E-13 | 5.9825E-11 |
| GO_LONG_CHAIN_FATTY_ACID_COA_LIGASE_ACTIVITY | -0.2407276 | 0.1299134 | -6.7989851 | 8.3105E-11 | 5.4019E-09 |
| GO_POSITIVE_REGULATION_OF_FATTY_ACID_BETA_OXIDATION | -0.2524632 | 0.14529912 | -5.3845065 | 1.7307E-07 | 7.7881E-06 |
| GO_FATTY_ACID_LIGASE_ACTIVITY | -0.2549822 | 0.17555802 | -6.7782609 | 9.3743E-11 | 5.9995E-09 |
| GO_CELLULAR_LIPID_CATABOLIC_PROCESS | -0.2710523 | 0.14144161 | -9.307761 | 8.6193E-18 | 8.2098E-16 |
| GO_REGULATION_OF_LIPID_CATABOLIC_PROCESS | -0.2900689 | 0.1153176 | -9.8919363 | 1.4388E-19 | 1.5396E-17 |
| GO_LIPID_CATABOLIC_PROCESS | -0.2921301 | 0.12354616 | -11.307303 | 4.8627E-24 | 5.8838E-22 |
| GO_FATTY_ACID_BETA_OXIDATION_USING_ACYL_COA_OXIDASE | -0.3116428 | 0.21631973 | -6.2419469 | 1.9458E-09 | 1.0702E-07 |
| GO_REGULATION_OF_FATTY_ACID_BETA_OXIDATION | -0.321115 | 0.16106982 | -7.4125987 | 2.1195E-12 | 1.5472E-10 |
| GO_FATTY_ACID_DERIVATIVE_CATABOLIC_PROCESS | -0.3384761 | 0.15073188 | -7.899509 | 1.0098E-13 | 7.8761E-12 |
| GO_NEGATIVE_REGULATION_OF_FATTY_ACID_BIOSYNTHETIC_PROCESS | -0.3613315 | 0.11447511 | -9.4237753 | 3.8564E-18 | 3.7793E-16 |
| GO_REGULATION_OF_FATTY_ACID_OXIDATION | -0.3666299 | 0.15752095 | -9.9849006 | 7.4311E-20 | 8.0999E-18 |
| GO_FATTY_ACID_BETA_OXIDATION | -0.3951864 | 0.21425436 | -8.9019021 | 1.386E-16 | 1.2751E-14 |
| GO_LIPID_OXIDATION | -0.3969457 | 0.19988518 | -9.7828792 | 3.1137E-19 | 3.2382E-17 |
| GO_FATTY_ACID_CATABOLIC_PROCESS | -0.4140073 | 0.21092358 | -9.8194193 | 2.405E-19 | 2.5252E-17 |
| GO_FATTY_ACID_BETA_OXIDATION_USING_ACYL_COA_DEHYDROGENASE | -0.5931341 | 0.27110943 | -9.4496945 | 3.2202E-18 | 3.2001E-16 |

Supplementary Table 4. General characteristics of the differently enriched Gene Oncology terms between low-risk group samples and normal hepatic samples with regard to TCGA

FC, fold change (low-risk samples vs. normal samples); AveExpr, average expression; adj, adjusted.
