# Supplementary material for: Construction of a lipid metabolism‐related and immune‐associated prognostic signature for hepatocellular carcinoma
Source: Cancer Med. 2020 Aug 19;9(20):7646–62. doi: 10.1002/cam4.3353 (PMC7571839; doi:10.1002/cam4.3353)
Supplement: Supplementary file 8 — Table S5 [file CAM4-9-7646-s008.docx]

Supplementary Table 5. Immune-related gene sets that associated with high-risk group

| Name | Size | ES | NES | NOM p-val | FDR q-val | FWER p-val |
| --- | --- | --- | --- | --- | --- | --- |
| GSE32986_UNSTIM_VS_CURDLAN_HIGHDOSE_STIM_DC_UP | 198 | 0.7009971 | 2.278759 | 0 | 0 | 0 |
| GSE40666_WT_VS_STAT1_KO_CD8_TCELL_WITH_IFNA_STIM_90MIN_DN | 199 | 0.724942 | 2.2738183 | 0 | 0 | 0 |
| GSE19941_LPS_VS_LPS_AND_IL10_STIM_IL10_KO_MACROPHAGE_UP | 194 | 0.7162793 | 2.2608416 | 0 | 0 | 0 |
| GSE7852_LN_VS_THYMUS_TCONV_DN | 200 | 0.7117012 | 2.2497962 | 0 | 0 | 0 |
| GSE10239_MEMORY_VS_KLRG1INT_EFF_CD8_TCELL_DN | 200 | 0.7167211 | 2.2484264 | 0 | 0 | 0 |
| GSE25085_FETAL_LIVER_VS_ADULT_BM_SP4_THYMIC_IMPLANT_DN | 200 | 0.7289907 | 2.2476025 | 0 | 0 | 0 |

ES, enrichment score; NES, normalized enrichment score; NOM, nominal; FDR, false discovery rate; FWER, familywise error rate.
