# Supplementary material for: Construction of a lipid metabolism‐related and immune‐associated prognostic signature for hepatocellular carcinoma
Source: Cancer Med. 2020 Aug 19;9(20):7646–62. doi: 10.1002/cam4.3353 (PMC7571839; doi:10.1002/cam4.3353)
Supplement: Supplementary file 9 — Table S6 [file CAM4-9-7646-s009.docx]

| Gene | Primer (5’→3’) |
| --- | --- |
| ACSL6 | F: GCTACACATATTCCATGGTG |
|  | R: TCTGAGGTTTGTCCACAATC 114bp |
| PLA2G1B |  |
|  | F: CTGTGCTGCTCACAGTGGCC |
|  | R: CACGCACTTGATCATTTTGCGG 83bp |
|  |  |
| SMPD4 | F: CCTCCCAGGACACCAGCCATACC |
|  | R: CGCTTTAGGAGGCTAGTGTGGTG 65bp |
| LPCAT1  LCAT  GAPDH |  |
|  | F: TCCTACTTCGACGCCATCCCTGT  R: AATCCTGGTCTGACCGGGACAC 136bp  F: CCTGGACAGCAGCAAGCTGGC  R: CGCCAGTCATAGGGGGCGGC 108bp  F 215: ATCCCATCACCATCTTCCAG  R 304: AGACGCCAGTGGACTCCACG 90b |

Supplementary Table 6. Primers of quantitative real-time polymerase chain reaction (qRT-PCR)

ACSL6, acyl-CoA synthetase long-chain family member 6; PLA2G1B, phospholipase A2 group 1B; SMPD4, sphingomyelin phosphodiesterase 4; LPCAT1, lysophosphatidylcholine acyltransferase 1; LCAT, lecithin-cholesterol acyltransferase; F, forward primer; R, reverse primer.
