# Supplementary material for: Construction of a lipid metabolism‐related and immune‐associated prognostic signature for hepatocellular carcinoma
Source: Cancer Med. 2020 Aug 19;9(20):7646–62. doi: 10.1002/cam4.3353 (PMC7571839; doi:10.1002/cam4.3353)
Supplement: Supplementary file 10 — Supplementary Materials [file CAM4-9-7646-s010.docx]

**Figure S1.** Differentially expressed transcription factors (TFs) with regard to TCGA. Heatmap (A) and volcano plot (B) illustrating differential TFs between HCC and non‐tumor tissues. In terms of the heatmap, the colors from green to red represent low to high gene expression levels. In the volcano plot, red dots represent differentially upregulated expressed genes, green dots represent differentially downregulated expressed genes, and black dots represent no differentially expressed genes. N, normal tissue. T, tumor.

**Figure S2.** Significantly enriched (A) cellular component GO terms and (B) molecular function GO terms.

**Figure S3.** The expression of (A) *LPCAT1*, (B) *PLA2G1B*, (C) *SMPD4*, (D) *LCAT*, and (E) *ACSL6* in each tissue.
